# Phosphorylation of PfiA modulates Pf4 phage production through PfiA/PfiT stoichiometric reconfiguration in *Pseudomonas aeruginosa*

**DOI:** 10.1126/sciadv.aeb5480

**Published:** 2026-04-03

**Authors:** Ran Chen, Yu Zhang, Yunxue Guo, Jiayu Gu, Shituan Lin, Xiaoxue Wang

**Affiliations:** ^1^ State Key Laboratory of Tropical Oceanography, South China Sea Institute of Oceanology, Chinese Academy of Sciences, Guangzhou 510301, China.; ^2^ Department of Ecology, College of Life Science and Technology, Jinan University, No. 601 Huangpu Avenue West, Guangzhou 510632, China.; ^3^ University of Chinese Academy of Sciences, Beijing 100049, China.

## Abstract

Filamentous Pf bacteriophages are widely distributed in *Pseudomonas aeruginosa* and profoundly influence biofilm formation and host virulence. The Pf4 prophage encodes a type II toxin-antitoxin (TA) system, PfiAT, modulating Pf4 propagation; however, its mechanistic role remains unclear. Here, through structural and biochemical analysis, we demonstrate that the PfiT toxin (ParE/RelE superfamily) has a unique C-terminal extension essential for TA complex formation. The antitoxin PfiA harbors a previously uncharacterized DNA binding domain, and its phosphorylation during biofilm formation shifts the PfiAT complex stoichiometry from a noncanonical PfiA_6_PfiT_4_ to a canonical PfiA_2_PfiT_2_ assembly. This phosphorylation is mediated by the prophage Pf6-encoded kinase toxin PfkA/PfkB at T5 in PfiA’s DNA binding domain. This posttranslational modification eliminates the pool of free toxins through complex reorganization, thereby neutralizing PfiT toxicity and enabling rapid Pf4 propagation during *P. aeruginosa* biofilm development. This study uncovers the cross-talk of TA systems from two coresident prophages and the role of posttranslational modification of TA system in mediating phage-phage and phage-host dynamics.

## Teaser

Antitoxin PfiA phosphorylation reconfigures PfiA/PfiT stoichiometry to modulate Pf4 phage production in *Pseudomonas aeruginosa*.

## INTRODUCTION

Filamentous phages belong to the family *Inoviridae* and the genus *Inovirus*, exhibiting a long filamentous morphology, with a genome consisting of circular single-stranded DNA (*1*–*4*). They are widely distributed in rivers, lakes, hot springs, soil, and host-associated environments such as plants and animals (*5*). These phages can either exist within host cells as plasmids (*6*) or integrate into the host genome at specific sites, replicating alongside the host genome (*7*). Unlike lytic phages, filamentous phages do not lyse their host cells upon infection. Instead, they use host resources to replicate their genomes and assemble and secrete phage particles at the host membrane, allowing the host cells to continue proliferating normally (*8*).

Pf phages, existing either as integrated prophages or episomes in the *Pseudomonas aeruginosa* genome, are activated during biofilm formation and subsequently secreted into the environment (*9*–*12*). Previous studies indicate that Pf4 prophage establishes a symbiotic relationship with its *P. aeruginosa* PAO1 host. The benefits of Pf4 production during biofilm formation and growth within eukaryotic hosts outweigh its cost to bacterial growth. These phages suppress mammalian immunity, aiding *P. aeruginosa* in evading clearance at infection sites (*13*). At high titers, Pf virions can align into liquid crystalline structures, shielding bacterial cells from antimicrobial penetration (*14*–*16*). The phage burden on host cells is also offset by enhanced biofilm robustness, provision of toxins or other virulence factors, and altered host motility (*7*, *15*). In addition, Pf phages confer superinfection exclusion by binding their capsid proteins (pVII and pIII) to type IV pilus (T4P) components (PilC and PilJ), thus defends against T4P-dependent lytic phages (*17*, *18*). A unique feature of biofilm-derived Pf4 phages is their ability to reinfect the same host cells, which is detrimental and can lead to host death (*9*, *19*, *20*). This complex symbiosis reflects a coevolutionary strategy where Pf phages enhance *P. aeruginosa* pathogenicity in exchange for stable replication and dispersal, thereby promoting antibiotic-resistant infections and complicating treatment.

Toxin-antitoxin (TA) systems are small genetic modules, ubiquitous in bacterial and archaeal genomes, encoding a stable toxin that disrupts essential cellular processes (such as DNA replication, translation, or cell wall synthesis) and a labile antitoxin (either a protein or a noncoding RNA) that counteracts the toxin’s activity (*21*). Their primary functions include stabilizing plasmids through “postsegregational killing” (eliminating daughter cells that lose the plasmid carrying the TA system) (*22*–*24*), defending against phage infection via abortive infection (*25*) and regulating biofilm formation and/or persister formation (*26*–*28*). TA systems are now classified into eight main types (I to VIII) based on the molecular nature of the antitoxin and its mechanism of toxin neutralization (*29*, *30*). In type II TA systems, both components are proteins, with the antitoxin directly binding to and inhibiting the toxin. Traditionally, toxin activation was thought to occur when the inherently unstable antitoxin within the TA complex is selectively degraded by intracellular proteases, thereby releasing the active toxin (*31*–*34*). Alternative mechanisms for toxin activation have also emerged, consistent with findings that some type II antitoxins are stably folded even without their cognate toxin (*35*). For example, in the absence of bacteriophages, the antitoxin AriA in the type II Paris TA system suppresses the tRNA^Lys^ cleavage activity of the toxin AriB through direct binding. Upon T7 phage infection, the phage-encoded Ocr protein binds to the AriA hexamer, releasing AriB, which subsequently forms a ribonuclease-active dimer. This dimer exerts toxicity by cleaving intracellular tRNA^Lys^ (*36*, *37*). In the *Mt*ParDE system, a temperature shift (from 4° to 37°C) in vitro induces the structural rearrangement of the ParD_2_ParE_2_ heterotetrameric complex into a ParD_4_ParE_2_ heterohexameric complex. During this reassembly process, two ParE molecules are released, which then act as the DNA gyrase inhibitor, thereby exhibiting toxic effects (*38*). Another strategy is exemplified by the plasmid-encoded HicA/HicB system, with the internal noncanonical transcription termination results in higher toxin production, enforcing plasmid maintenance through a postsegregational killing “addiction” mechanism within microbial communities (*39*).

*P. aeruginosa* MPAO1 strain carries two coresident filamentous prophages, Pf4 and Pf6. They share a very conserved core region encoding for phage structural protein and assembly, while both also carry unique TA systems (*40*). Pf6’s accessory KKP TA module (kinase-kinase-phosphatase, PfkA-PfkB-PfpC) governs Pf4 and Pf6 induction during attached biofilm formation using the flow-cell biofilm setting, through reversible phosphorylating host silencer MvaU protein (*40*). Recently, we found that one of the core proteins of Pf4, RepG4 (PA0717), triggers kinase-mediated toxicity of KKP in a dose-dependent manner by degrading the phosphatase antitoxin (*41*). Pf4 encodes a type II TA system PfiT/PfiA, and deleting the *pfiT* toxin gene promotes the formation of superinfective phages during pellicle biofilm development using statically growing conditions (*42*). However, the regulatory mechanisms by which the PfiAT system controls Pf phage production remain largely unclear. In addition, the relationship between the PfiAT system of Pf4 and the KKP TA system of Pf6 is unknown. In this study, we found that phosphorylation of the antitoxin PfiA during the late stage of biofilm formation triggers a shift in the TA stoichiometry from a 6:4 assembly to a 2:2 assembly. The formation of the original 6:4 assembly inherently generates a pool of free PfiT toxin responsible for low-level toxicity, whereas the newly formed 2:2 assembly efficiently sequesters these free toxins. This reduction in free toxin levels diminishes cellular toxicity and enhances Pf4 phage production, revealing a unique paradigm for TA regulation.

## RESULTS

### The C-terminal extension of PfiT plays a pivotal role in Pf phage production in biofilm

**Fig. 1.**
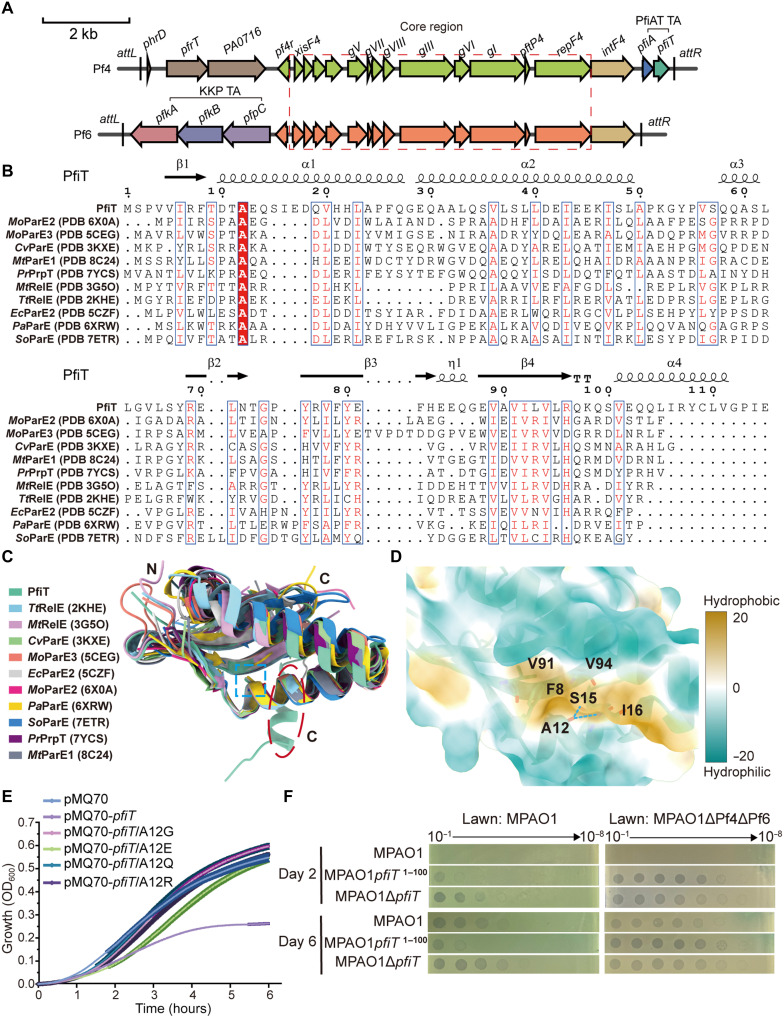
The C-terminal region of PfiT plays a pivotal role in regulating the lysogeny of the Pf4 phage. (**A**) Annotation of Pf4 and Pf6 phages. The core region is highlighted with a red dashed box, and the PfiAT and KKP systems were labeled. (**B**) Multiple sequence alignment of PfiT with orthologs and other published structures. The secondary structure elements were labeled above the sequences. (**C**) Structural superimposition of the backbone structure of PfiT (colored medium aquamarine) onto that of *Tt*RelE from *T. thermophilus* (PDB 2KHE, cyan), *Mt*RelE from *M. tuberculosis* (PDB 3G5O, light purple), *Cv*ParE from *C. vibrioides* (PDB 3KXE, green), *Ec*ParE2 from *E. coli* (PDB 5CZF, gray), *Mo*PraE2 (PDB 6X0A, magenta) and *Mo*PraE3 (PDB 5CEG, light red) from *M. opportunistum*, *Pa*ParE from *P. aeruginosa* (PDB 6XRW, yellow), *So*ParE from *S. oneidensis* (PDB 7ETR, blue), *Pr*PrpT from *P. rubra* (PDB 7YCS, purple), and *Mt*ParE1 from *M. tuberculosis* (PDB 8C24, dark gray). Differences in the C terminus of PfiT are highlighted with red dashed ellipses, and the conserved alanine residues are labeled with blue dashed rectangles. (**D**) The interactions involving the Ala^12^ residue of PfiT. (**E**) Growth curves of WT and Ala^12^-mutated PfiT. (**F**) Phage titers of day 2 and 6 biofilm effluents from the indicated MPAO1 and *pfiT* deletion strains plated on lawns of the two different host strains. Three independent replicates were performed, and only representative images are shown here.

PfiT toxin in Pf4 prophage belongs to the RelE/ParE superfamily (PF05016) (Fig. 1A). Ten previously studied homologs that have the closest structure resemblance to PfiT were identified using AlphaFold 3 for Dali server searches, including the *P. aeruginosa* PAO1 chromosomal-encoded ParE [Protein Data Bank (PDB) 6XRW] (*43*), the ParE2 from *Escherichia coli* O157: H157 (PDB 5CZF) (*44*), the PrpT toxin from the megaplasmid of *Pseudoalteromonas rubra* (PDB 7YCS) (*45*), PraE2 (PDB 6X0A) (*46*) and PraE3 (PDB 5CEG) from *Mesorhizobium opportunistum* (*47*), ParE from *Caulobacter vibrioides* (PDB 3KXE) (*48*), RelE from *Mycobacterium tuberculosis* (PDB 3G5O) (*49*), RelE from *Thermus thermophilus* (PDB 2KHE), ParE from *Shewanella oneidensis* (PDB 7ETR) (*50*), and ParE1 from *M. tuberculosis* (PDB 8C24) (*38*). These proteins share low sequence identity, with only one alanine residues conserved across all 11 members, yet their overall structures exhibit high similarity [root mean square deviation (RMSD) range: 2.5 to 5.2 Å over 91 to 94 Cα atoms] (Fig. 1, B and C). We then characterized the conserved A12 residue located at the α1 helix N terminus. Structural data show that A12’s carbonyl oxygen forms hydrogen bonds with S15 and I16, while its side chain inserts into a hydrophobic pocket formed by F8, I16, V91, and V94 (Fig. 1D), suggesting a stabilizing role. Alanine-to-glycine/glutamate/arginine/glutamine substitutions all abolished PfiT toxicity (Fig. 1E and fig. S1A), confirming that A12-mediated hydrophobic interactions are essential. Phenotypically, these mutations resulted in reduced filamentous growth and cell lysis (fig. S1B).

Notably, the PfiT is the longest, having an additional α helix composed of 15 amino acids at the C terminus (Fig. 1B). Structural alignment revealed divergent regions in PfiT, with the most notable feature being a spatially distinct α helix formed by its C-terminal extension (Val101-Val111) (Fig. 1C). To further elucidate how the PfiAT system regulates Pf phage production, we examined Δ*pfiT* (full deletion) and pfiT^1–100^ (C-terminal truncation) mutant strains in our flow-cell biofilm system, which recapitulates chronic infection conditions through biofilm formation on catheter surfaces (*40*). Analysis of biofilm effluents from days 2 to 6 showed that while wild-type (WT) MPAO1 exhibited gradual increases in superinfective phage production (gradually increase from day 2 to day 6), both Δ*pfiT* and the truncated mutants produced substantial quantities of Pf phages as early as day 2 (Fig. 1F). These results demonstrate that the C-terminal extension of PfiT plays a crucial role in modulating the production of superinfective Pf4 phages during biofilm development.

### Crystal structure of the PfiA_6_PfiT_4_ complex

**Fig. 2.**
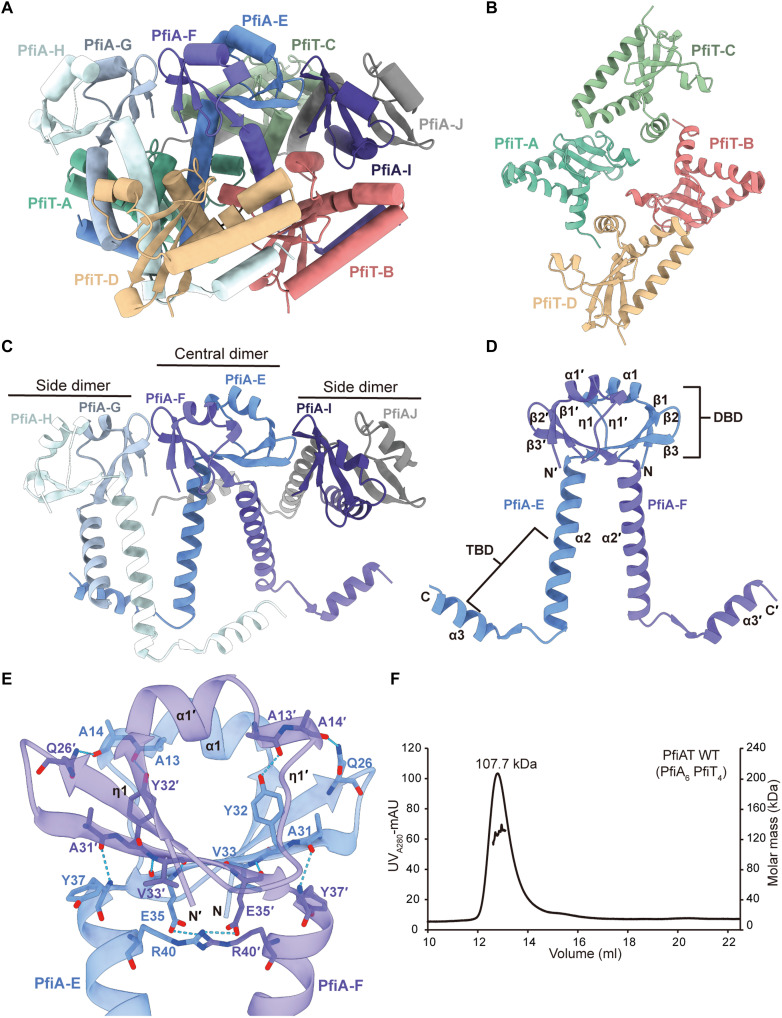
PfiA and PfiT form a complex in a 6:4 ratio. (**A**) The PfiAT decamers structure shown in the cylinder rendition views. (**B**) The four PfiT monomers and (**C**) three PfiA dimers in the decamers complex were shown in the cylinder rendition views. (**D**) The PfiA dimeric structure located in the middle of the complex shown in the ribbon rendering views. The DNA binding domain (DBD) and toxin binding domain (TBD) of PfiA were labeled. (**E**) Close-up view of the interactions between chain E and chain F homodimer of PfiA. (**F**) Molecular weight of the PfiAT complex determined by SEC-RALS. A molecular weight of 107.7 kDa is consistent with a PfiA:PfiT complex in a 6:4 stoichiometric ratio.

The crystal structure of the PfiA-PfiT complex was resolved to further explore how PfiAT regulates phage production (Fig. 2A). The coexpressed complex displayed a purity of ~95% following the removal of the N-terminal 6× His-tag. The crystals exhibited diffraction patterns with a resolution of 2.7 Å, and the highest quality crystal diffracted to 2.3 Å with 99.4% completeness. The final model incorporates 165 water molecules. All residues are in favorable geometric conformations, with no residues falling into the Ramachandran outlier region (table S1). The asymmetric unit contains a large TA complex comprising four PfiT chains (A to D) and six PfiA chains (E to J), forming a decameric assembly (Fig. 2, B and C). The electron density maps allowed unambiguous tracing of PfiT residues S2-E115 (chain A), P3-P113 (chain B), S2-G112 (chain C), and P3-G112 (chain D). For PfiA, continuous density was observed for M1-F79/A80/P83 (chains E, F, J, and H) and M1-A54 (chains G and I). Regions with poor electron density (L16-L18 and Q26-G28 in chain H; S19 in chain G) were excluded from the final model (Fig. 2C).

In the PfiAT decamer complex, six PfiA molecules form three PfiA homodimers, with the homodimer composed of chains E and F adopting a “W” shape, composing a fundamental building block of the PfiAT complex (Fig. 2D). These two monomers exhibit a high degree of similarity, as evidenced by an RMSD of ~0.47 Å for 79 Cα atoms upon superposition. Each monomer consists of three helices and three additional β strands, with the six β strands from the two monomers forming a β sheet (fig. S2, A and B). The DNA binding domain is comprised of α1, η1, and β1-3, while the toxin binding domain consists of α2 and α3. The interaction between chains E and F primarily occurs at the DNA binding domain of PfiA antitoxins, forming 11 pairs of hydrogen bonds and a salt bridge. Specifically, the backbone carbonyl group of A13 and A14 on chain E accepts a hydrogen bond from Y32 and Q26 side chain on chain F, respectively. The backbone of chain E/E35 forms two hydrogen bonds with the backbone of chain F/V33, while the side chain forms a salt bridge with chain F/R40. The backbone amino group of chain E/Y37 donates one hydrogen bond to chain F/A31. Because the distribution of interacting residues has twofold rotational symmetry, equivalent residues (A13, A14, A31, E35, and Y37) on both chains form symmetrically paired hydrogen bonds and a salt bridge with adjacent residues (Q26, A31, Y32, V33, and R40) through either backbone or side chain interactions (Fig. 2E). In short, of the 12 pairs of interactions formed between chains E and F, 8 are contributed by residues on the antiparallel β3 strands of chains E and F, while the remaining 4 pairs are formed by residues in the β3 and the N-terminal region of α2 in both chains, with barely contact at the C termini of the two chains.

Upon superimposing the three dimers of PfiA antitoxins, it was observed that the conformations of full-length chains E, H, and J on one side were nearly identical. In contrast, chains G and I, lacking electron density at the C-terminal end, exhibited perfect alignment, with their α2 helix inwardly deflected by ~25° compared to full-length chain F (fig. S2C). We hypothesize that if the C termini of the G and I chains adopt the same conformation as the rest of the four chains, the C-terminal end of α2 would directly clash with the other chain in the dimer. To examine potential proteolytic degradation of chains G and I during protein expression or crystallization, five PfiAT crystals were subjected to three sequential washes in cryoprotectant solution. Following this, the crystals were dissolved in water for matrix-assisted laser desorption/ionization–time-of-flight (MALDI-TOF) mass spectrometry analysis. The results revealed two predominant molecular weights within the sample: 13,224.6 Da, corresponding to the full-length PfiT (13,227 Da), and 9443.6 Da, corresponding to the full-length PfiA (9444 Da). This indicates that the missing C termini of chains G and I in the model exist in a disordered conformation due to the absence of binding with PfiT (fig. S2D). Furthermore, size exclusion chromatography coupled to right-angle light scattering (SEC-RALS) experiments were used to determine the molecular weight and stoichiometry of the TA complex. The molecular weight of the PfiA-PfiT complex was determined to be ~107.7 kDa (Fig. 2F), indicating that the PfiA-PfiT complex exists in solution as a decamer. Within the same TA family, ParD_4_ParE_2_ captured by recent crystal structures was 59 kDa (*38*), and the ParD_6_ParE_2_ discovered through native mass spectrometry was 78 kDa (*51*). To our best knowledge, the PfiA_6_PfiT_4_ complex is the largest among the now known binary ParDE family members.

### The C-terminal extension of the PfiT is critical for TA complex assembly

**Fig. 3.**
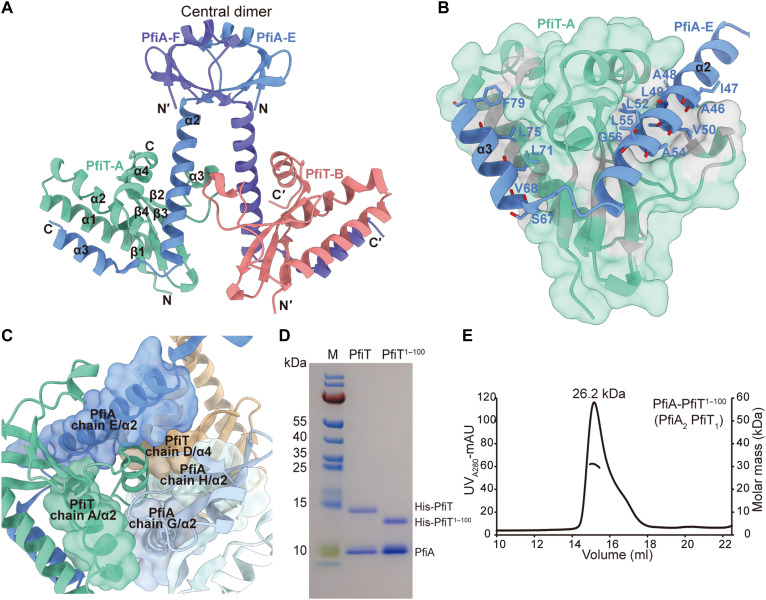
The C-terminal extension of the PfiT is critical for TA complex assembly. (**A**) The structure of PfiT interacting with the centrally positioned PfiA dimer. (**B**) The hydrophobic interactions at the interface between PfiT/chain A and PfiA/chain E. (**C**) Close-up view of the environment surrounding the C-terminal of PfiT. The five α helices involved in the interaction are displayed as surfaces. (**D**) Coexpression and nickel–nitrilotriacetic acid (Ni-NTA) purification of the PfiAT complex, as visualized by SDS–polyacrylamide gel electrophoresis (SDS-PAGE) electrophoresis. (**E**) Molecular weight of the PfiA-PfiT C-terminal truncated complex determined by SEC-RALS.

Among the three dimers formed by PfiA, only chains G and I, lacking the C-terminal region, did not bind to PfiT. The other four PfiA chains each bound to one PfiT monomer. The centrally positioned PfiA-E and PfiA-F chains engage in interactions with PfiT-A and PfiT-B, respectively, exhibiting a binding pattern that closely resembles the canonical 2:2 complex architecture observed in ParDE systems (Fig. 3A) (*38*, *43*, *50*). For our analysis, we selected PfiA/chain E and PfiT/chain A as representative complexes (Fig. 3B). In the heterodimer, PfiT establishes extensive contact with the V-shaped inner surface formed by the toxin binding domain of PfiA, resulting in a buried surface area of 1669 Å^2^, larger than that of the PfiA dimer (985 Å^2^) (Fig. 3B). However, PfiT has minimal contact with the DNA binding domain of PfiA. Structurally, PfiT consists of four α helices and four β strands, forming a relatively compact configuration. The PfiT surface involved in the interaction with PfiA features two hydrophobic regions. The larger hydrophobic region comprises residues Q59, A60, L63, V65, F79, L93, A90, S100, Q104, and L105, which engage in hydrophobic interactions with the A46-G56 segment on α2 of PfiA/chain E. The smaller hydrophobic surfaces consist of T9, I6, I16, V20, H21, L33, L37, L40, H83, and I92, which interact hydrophobically with S67, V68, L71, L75, and F79 on α3 of PfiA/chain E (Fig. 3B). In addition, there are a lot of hydrogen bonds and salt bridges between PfiT and PfiA in the α2, α3, and loop between α2 and α3 (fig. S3, A and B). Therefore, we reasoned that PfiA may neutralize the toxicity of PfiT by preventing its dimerization or inhibiting its interaction with other target molecules.

Within the complex, a barrel-like structure is formed by the five α helices from chain A/α2, chain D/α4, chain E/α2, chain H/α2, and chain G/α2, with the C-terminal α4 of chain A acting as a plug inserted into the cavity surrounded by these five helices (Fig. 3C). The C terminus of chain A engages in six pairs of hydrogen bonds with the adjacent α helices, while the additional α3 region interacts with another toxin, forming two pairs of hydrogen bonds (fig. S3, C and D). The C-terminal α4 of chain A aligns in a cis-parallel orientation with chain D’s α4, devoid of polar interactions but featuring extensive hydrophobic interactions. Notably, the lone cysteine residue, C109, in the two proteins of the PfiAT system, is located within PfiT’s α4 helix, engaging in hydrophobic interactions. These cysteines are positioned closely with their side chains facing each other, with a thiol-thiol distance of 3.6 Å, likely forming a physiological-state covalent disulfide bond (fig. S4A). To assess disulfide bond formation in the PfiAT complex, we performed redox analysis. SDS–polyacrylamide gel electrophoresis (SDS-PAGE) showed covalent dimers in untreated samples, a proportion unaffected by oxidized glutathione, indicating spontaneous and efficient disulfide bond formation during purification. These dimers were eliminated by reduced glutathione, confirming their disulfide linkage (fig. S4B). In addition, the central antitoxin dimer is crucial for assembly, as chain E interacts with multiple toxin chains including A, B, and D (fig. S4C). To evaluate the importance of the C-terminal extension of PfiT, full-length PfiA was coexpressed and purified with a truncated form of the C-terminal region of PfiT (PfiT^1–100^). Despite the truncation of extra α4, PfiT still interacted with PfiA (Fig. 3D). However, SEC-RALS analysis of TA complex revealed a marked reduction of size from 107.7 kDa with PfiT to 26.2 kDa with PfiT^1–100^, resulting in a rare A:T ratio of 6:4 to a canonical ratio of 2:1 of the TA family (Fig. 3E). These results collectively showed that the addition of a unique extension of C terminus of ParE toxin family altered TA complex assembly.

### The N terminus of PfiA features a previously uncharacterized DNA binding domain

**Fig. 4.**
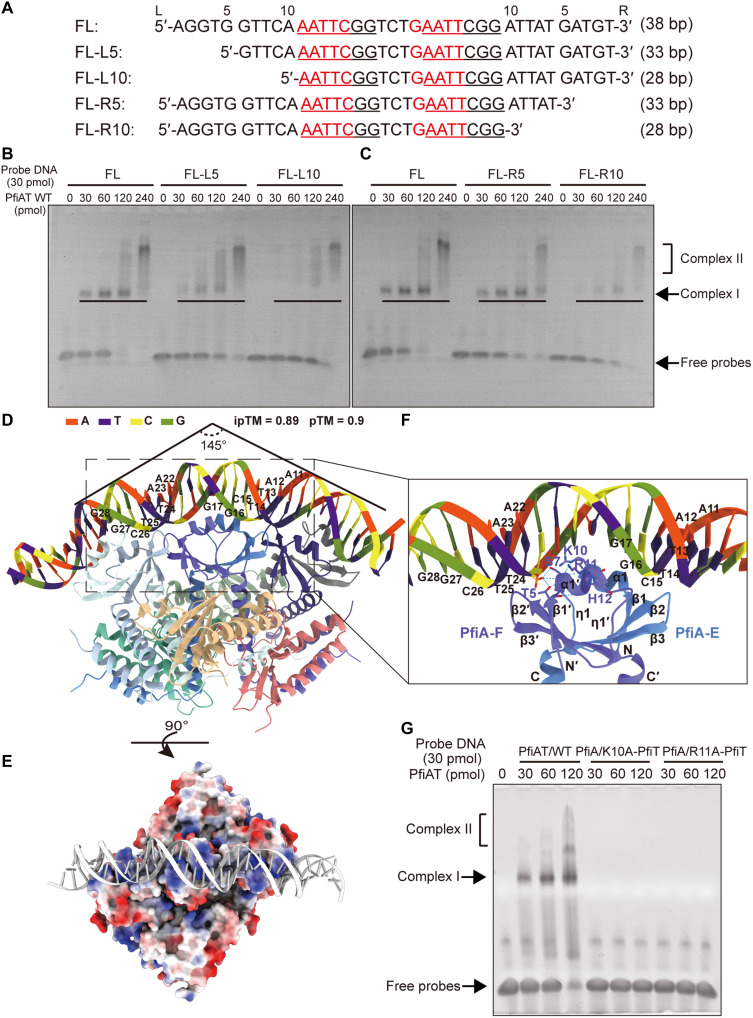
The N terminus of PfiA represents a previously uncharacterized DNA binding domain. (**A**) DNA sequences of varying lengths used in the EMSA assay. The red regions indicate pseudo-palindromic sequences, and underlined regions denote repetitive sequences. L (left) and R (right) denote the directionality of the flanking sequences. (**B** and **C**) EMSA analysis of the binding affinity of the WT PfiAT complex. The molar ratios of protein to DNA are indicated above the gel, while the bands corresponding to free DNA and protein-bound DNA complexes are shown on the left. The black solid line represents the decreasing concentration of complex I. (**D**) Structural model of the PfiAT–38-bp DNA complex predicted using AlphaFold 3. The predicted ipTM and pTM values of the complex structure are labeled accordingly. (**E**) Surface charge analysis of the PfiAT complex, calculated using Adaptive Poisson-Boltzmann Solver (APBS). Blue represents positive charges, and red represents negative charges. (**F**) Analysis of the interaction interface between the central PfiA dimer and DNA. (**G**) Analysis of the DNA binding affinity of PfiA K10A and R11A mutants in the PfiAT complex by EMSA.

Our previous study demonstrated that the PfiAT complex binds to a pseudo-palindromic sequence 5′-AATTC-N_5−_GAATT-3′ in its own promoter region. However, unlike most canonical type II TA systems, the antitoxin PfiA alone lacks binding affinity for this region (*42*). We observed that, in the *pfiAT* promoter region, the pseudo-palindromic sequences “AATTC” are also part of the “AATTCGG” repeat sequences (fig. S5A). Next, a 38-bp promoter DNA segment containing the repeats and pseudo-palindrome (referred to as 38-bp FL) with the 5′ and 3′ end-truncated segments (referred as FL-L/R5 and FL-L/R10) were synthesized and used as DNA probes for electrophoretic mobility shift assay (EMSA) with PfiAT (Fig. 4A). A specific complex, complex I, was observed at protein-to-DNA ratios of 1:1 and 2:1. A second complex, complex II, appeared with increasing protein concentration at a ratio of 8:1. When using truncated DNA, the formation of retarded bands by FL-L5 and FL-R5 was notably reduced compared to the 38-bp FL. In the reaction involving FL-R10, complex I persisted, while it disappeared entirely when replaced with the FL-L10 DNA segment (Fig. 4, B and C). This indicates the presence of a third recognition region for the PfiAT complex within the upstream region of the repetitive sequence. In experiments using C-terminally truncated PfiAT complex with a T:A ratio of 1:2 was unable to bind to any DNA sequences (fig. S5, B and C). These findings imply that PfiAT binding to the promoter relies on the assembled TA decamer, with three PfiA dimers actively participating in DNA binding.

The recent advances in AlphaFold 3 have provided previously unexplored tools for predicting protein–nucleic acid complex structures, which we used to construct a structural model of the PfiAT complex bound to promoter DNA (Fig. 4D). The predicted ternary complex model shows that the DNA is bent by ~145°, with all three PfiA dimers interacting with the DNA fragment (Fig. 4D). Electrostatic potential analysis reveals extensive positively charged patches at the binding interfaces between the three dimers and DNA (Fig. 4E). The first 36 amino acids of PfiA form a β1-α1-η1-β2-β3 folding pattern (Fig. 2E and fig. S6, A and B). Dimerization is mediated by antiparallel interactions between α1 and β1 from two monomers, a configuration also found in antitoxins of type II TA systems such as RelB (PDB 3G5O) (*49*), Phd (PDB 4ZLX) (*52*), and YefM (PDB 6L8E, 7V6W, and 8YXV) (*53*–*55*), previously classified as HTH or wHTH domains (fig. S6, B and C). A unique feature is that PfiA contains an η helix between α1 and β2 instead of the α helix present in other members (Fig. 4F and fig. S6C). The three PfiA dimers each insert into three adjacent major grooves of DNA, with two α1 helices aligned parallel to the DNA helical axis on either side, while the loop region between β2-β3 embeds into the minor groove (Fig. 4F). This binding mode shows high similarity to the previously reported structures of Phd-DNA (PDB 4ZM0) (*52*) and YoeB-YefM-DNA complexes (PDB 6L8E and 7V6W) (fig. S6, D and E) (*53*, *54*). Unlike classical 2:2 TA complex, the 6:4 assembly creates a larger DNA binding interface, offering greater regulatory plasticity. Notably, the three PfiA dimers in this complex bind to three distinct regions of *pfiAT* promoter that contains tandem and direct repeats in a precisely defined spatial arrangement. This multivalent binding to repetitive elements underpins the complex’s heightened stability and specificity in transcriptional repression. Analysis of the AlphaFold 3–predicted PfiAT-DNA interface revealed contacts between T5/S7 and the phosphate backbone but no involvement of the basic residues K10/R11 on the α1 helix. Notably, PfiA lacks residues capable of specific nucleobase recognition in the pseudo-palindromic sequence, indicating limitations in the current model. Individual alanine mutations at K10 and R11 abolished DNA binding in EMSA assays (Fig. 4, F and G), confirming their essential role in PfiAT-DNA binding.

Compared to the HTH domain commonly found in DNA binding antitoxins, we observed substantial differences in the secondary structure composition, dimerization mechanism, and DNA binding mode of PfiA’s N-terminal structural domain. The HTH domain, as the most prevalent DNA binding domain in prokaryotic and some eukaryotic genomes, is primarily found in transcription factors. Beyond transcriptional regulation, these proteins play crucial roles in DNA repair, replication, RNA metabolism, and protein-protein interactions in signal transduction. This domain can be divided into 11 subtypes, with the typical HTH type consisting of three α helices forming the core structure, while other types may contain additional one to three α helices or two to four β strands (*56*). These proteins typically function as dimers, but direct interactions between HTH domains are not essential. For example, in the MqsRA TA system, the DNA binding domain at the C terminus of the antitoxin MqsA contains three α helices (α2 to α4) of the HTH domain (fig. S6F), with dimerization mediated by interactions between α5 and α6 independent of the HTH domain. Similar to other HTH proteins, MqsA binds DNA by inserting the third α helix of its HTH domain into the major groove (fig. S6F) (*57*). These results suggest that the DNA binding domain exhibited by PfiA should be classified as a separate domain. As it has only been found at the N terminus of antitoxin proteins, we propose to name it the PAD (PfiA-like antitoxin DNA binding) domain.

### T5 phosphorylation of PfiA shifts the PfiA:PfiT stoichiometry and abolishes DNA binding

**Fig. 5.**
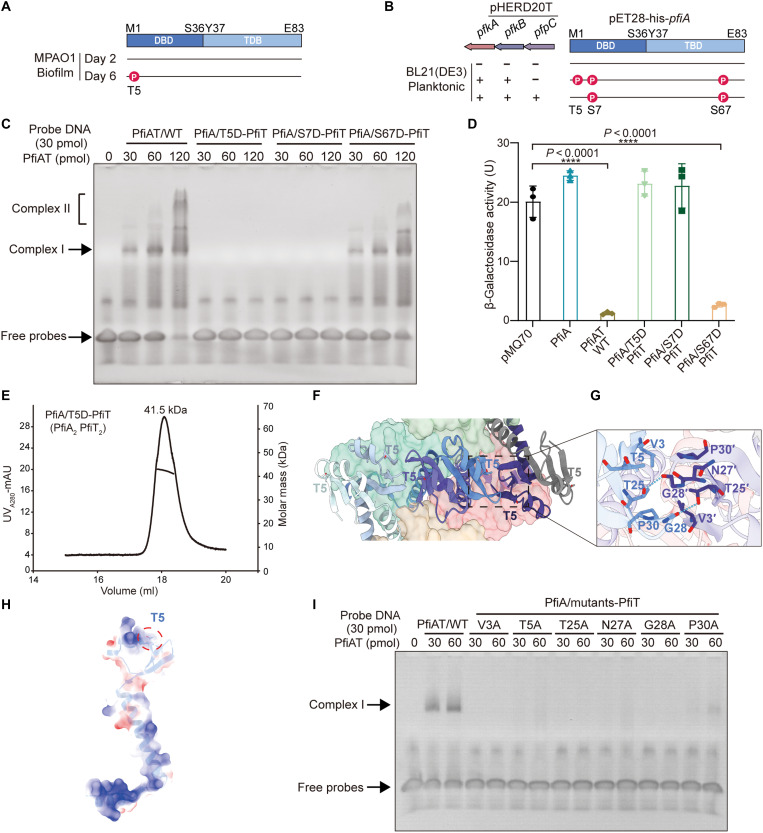
Phosphorylating T5 of PfiA alters the stoichiometry of PfiA to PfiT in the complex. (**A**) Phosphoproteomic profiling of MPAO1 strains grown in flow-cell biofilm systems at day 2 and day 6. (**B**) Mass spectrometric analysis of phosphorylated PfiA coexpressed with PfkA/PfkB kinase in *E. coli* BL21. The mass spectrometry–identified phosphopeptide data are presented in fig. S5 and tables S4 and S5. (**C**) EMSA experiments to evaluate the DNA binding capability of the phosphorylation-mimicking PfiAT complex. (**D**) The promoter activities of the mutated PfiAT were determined in strains MPAO1Δ*pfiAT*. Three independent cultures of each strain were used, and error bars indicate SD. ****P < 0.0001. (**E**) Molecular weight determination of the PfiA/T5D-PfiT mutant complex using SEC-RALS. (**F**) Spatial distribution of T5 across the six monomers of PfiA. (**G**) Interactions between the DNA binding domains of two adjacent dimers. Two pairs of hydrogen bonds are indicated by dashed lines, and residues involved in hydrophobic interactions with T5 are shown in stick representation. (**H**) Surface charge analysis of the PfiA monomer, with positively charged regions surrounding T5 highlighted by a red circle. Blue represents positive charges, and red represents negative charges. (**I**) EMSA analysis of the DNA binding ability of complexes with mutations at different sites.

Previous studies investigating how the KKP system regulates Pf prophage lysogeny performed phosphoproteomic analysis of MPAO1 biofilm cells to identify potential PfkA/PfkB kinase targets (*40*). Alongside phosphorylated proteins involved in fundamental cellular processes, including cell division, DNA replication, transcription, translation, and stress response, phosphorylation of PfiA at T5 was detected in biofilm cells producing superinfective phages at day 6 (Fig. 5A). To exclude potential interference from endogenous genes in the MPAO1 strain, we used a dual-plasmid system to overexpress KKP and His-tagged PfiA in *E. coli* BL21. Cells were harvested 3 hours after dual inducer treatment, and PfiA was extracted for phosphoproteomic analysis. In strains expressing PfkA/PfkB, phosphorylation of PfiA at T5, S7, and S67 was detected, whereas in samples expressing all three KKP components, phosphorylation at S7 and S67 was still observed. No phosphorylation at any site of PfiA was detected in the empty vector control (Fig. 5B and fig. S7, A to C).

Among the phosphorylation sites identified in PfiA, T5 and S7 reside within the PAD domain and participate in DNA interactions (Fig. 4F), leading us to hypothesize that their phosphorylation may impair the DNA binding capacity of the PfiAT complex. To test this, we compared the DNA binding activities of the WT PfiAT complex with those of phosphomimetic mutants (T5D, S7D, and S67D) using EMSA experiments. The results showed that the T5D and S7D mutants, but not S67D, completely lost DNA binding ability (Fig. 5C). We also check their abilities to repress the *pfiTA* promoter activity in the MPAO1Δ*pfiAT* strain. Consistently, the TA complex with T5D or S7D mutant lost its ability to repress the promoter activity, while the TA complex with S67D retained this repression activity (Fig. 5D). In addition, SEC-RALS analysis confirmed that the T5D mutant formed a 41.5-kDa complex, consistent with a PfiA_2_PfiT_2_ stoichiometry (Fig. 5E). In contrast, both S7D and S67D exhibited molecular weights close to the WT complex, corresponding to a PfiA_6_PfiT_4_ assembly (fig. S8A). These results demonstrate that phosphorylation of T5 by PfkA/PfkB not only introduces local electrostatic alterations but also fundamentally shifts the PfiAT assembly from a 6:4 to a 2:2 stoichiometry. The loss of DNA binding capacity in the T5D mutant phenocopied the C-terminally truncated PfiT complex (PfiA_2_PfiT_1_), indicating that both the C-terminal extension of PfiT and the DNA binding domain of PfiA are essential prerequisites for the assembly and stability of the native 6:4 complex.

To understand how phosphorylation of T5 affects the PfiAT ratio fundamentally, we checked the positioning of the T5 in the complex, where the T5 of the six PfiA monomers in the complex are arranged in a wavy pattern (Fig. 5F). Unlike solvent-exposed S7/S67 (fig. S8, B and C), T5 localization is chain dependent and solvent accessible in peripheral chains H and G but embedded at dimer interfaces in internal chains E, F, I, and J. Analysis of the E-F/G-J dimer interface reveals a 253-Å^2^ contact area, featuring two reciprocal hydrogen bonds (chain E-T25/chain J-G28 and chain E-G28/chain J-T25) and hydrophobic stacking between chain E-V3/T5/P30 and chain J-V3/A27/P30 (Fig. 5G). As the T5 residue is in a positively charged region, phosphorylation by PfkA/PfkB introducing negative charges may lead to local charge rearrangement (Fig. 5H). To further distinguish whether the negative charge introduced by phosphorylation of PfiA directly affects its interaction with DNA or disrupts the hydrophobic interaction network involving T5 postphosphorylation, we generated mutations of T5 and other residues involved in interactions to alanine. EMSA experiments revealed that mutations at all sites, except P30A, completely lost their DNA binding ability, with P30A retaining a weak binding affinity to DNA (Fig. 5I). The results show that DNA binding by the PfiAT complex depends on the formation of a 6:4 stoichiometric assembly, reinforcing its ratio-dependent mode of transcriptional regulation.

### The T:A ratio of the PfiAT complex regulates Pf4 prophage induction

**Fig. 6.**
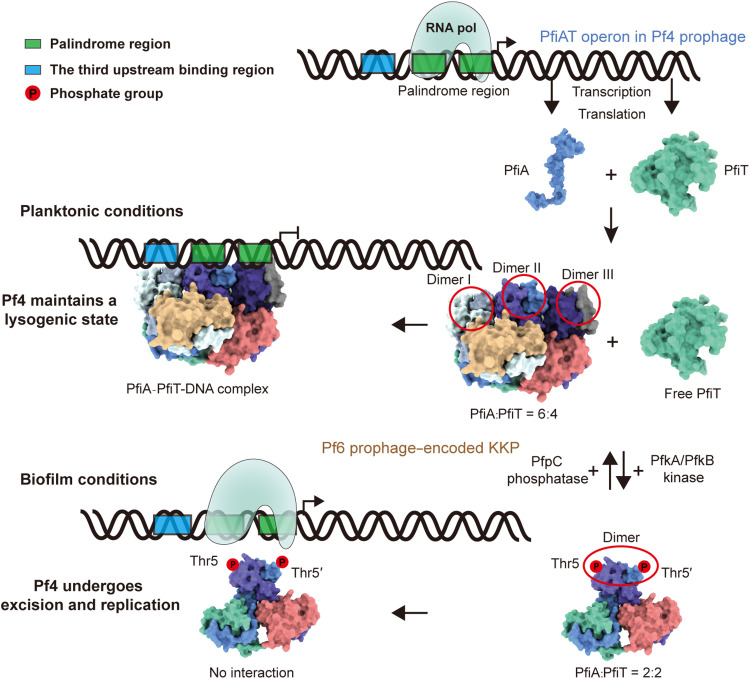
Schematic model of the regulation of the PfiAT operon. In planktonic conditions, the PfiA-PfiT complex binds promoter DNA in a 6:4 stoichiometry to autoregulate its own transcription, while free PfiT acts through a postsegregational killing–like mechanism to inhibit prophage excision, thereby stabilizing the lysogenic state. Under biofilm induction, PfkA/PfkB-mediated phosphorylation of PfiA triggers reorganization of the complex into a 2:2 form that cannot bind DNA, thereby derepressing *pfiA*/*pfiT* expression. In the absence of free toxin, this leads to prophage activation.

This study elucidates the molecular mechanism by which the PfiAT system regulates the lysogenic maintenance of Pf4 phage. By integrating biochemical and structural evidence from current and previous studies, we propose a regulatory model for PfiA-PfiT (Fig. 6). Under planktonic growth conditions, transcription and translation of the Pf4-encoded *pfiA* and *pfiT* genes yield a PfiA_6_PfiT_4_ complex at an optimal stoichiometric ratio. Within this complex, two PfiA dimers bind the pseudo-palindromic sequence in the promoter region, while the third dimer occupies the 5′ end of the pseudo-palindrome, thereby suppressing *pfiA*/*pfiT* transcription (notably, PfiA alone lacks this repressive activity) (*42*). The *pfiA* and *pfiT* genes are cotranscribed and are expected to produce proteins at similar levels, as they also share the identical ribosome-binding site (fig. S4A). Formation of the 6:4 complex generates free cytoplasmic PfiT, a fraction of which uses a postsegregational killing–like mechanism, analogous to the plasmid-stabilizing TA system, to inhibit Pf4 phage excision during lysogeny.

Under biofilm conditions, PfpC (the antitoxin of the tripartite KKP TA system encoded by Pf6) interacts with RepG4 and undergoes degradation (*41*). This activates the toxin components PfkA/PfkB, which phosphorylate T5 of PfiA. This posttranslational modification disrupts interdimeric PfiA interactions, driving the formation of a PfiA_2_PfiT_2_ complex. The stoichiometric shift from 6:4 to 2:2 requires recruitment of free PfiT, depleting the cytoplasmic PfiT pool and ultimately triggering Pf4 phage excision and virion production. Concurrently, the reorganized complex loses promoter DNA binding capacity, enabling de novo transcription/translation of PfiA and PfiT. Thus, KKP-mediated phosphorylation precisely controls the PfiAT stoichiometric balance. This toxin-release mechanism fundamentally differs from classical models wherein antitoxin instability and proteolysis by Lon and/or ClpXP protease complexes govern toxin liberation.

## DISCUSSION

This study investigated the molecular mechanism by which the PfiAT TA system encoded by the Pf4 prophage of *P. aeruginosa* regulates Pf4 phage propagation during biofilm formation. PfiAT belongs to type II TA system, but it also exhibits several unique features: (i) The antitoxin PfiA lacks DNA binding ability when present alone, which differs from most type II TA systems; (ii) system regulation depends on changes in stoichiometric ratios rather than antitoxin degradation; (iii) it forms a cross-regulatory network with another prophage-encoded KKP system. Cross-talk between different types of TA systems has been documented in various bacterial hosts. For instance, in *E. coli* K-12 biofilms, the type II TA MqsRA regulates the type V TA GhoTS via MqsR toxin–mediated, sequence-dependent cleavage of the *ghoS* antitoxin mRNA (*58*) and controls the rac prophage–encoded type I TA RalRA (*59*). ParEso of prophage CP4So of *Shewanella onidensis* plays a critical role in the maintenance of CP4So in host cells after its excision, and the cognate antitoxin CopA_SO_ cross-regulates the megaplasmid-encoded PemK/PemI system by targeting homologous promoter motifs (*60*). Here, we report analogous cross-talk during *P. aeruginosa* MPAO1 biofilm formation: The Pf6 prophage–encoded type VII KKP system regulates the Pf4 prophage–encoded type II TA PfiAT. Specifically, the Ser/Thr type of kinase toxin mediates posttranslational modification of the PfiA antitoxin, thereby modulating the PfiT toxin pool. While we previously identified interactions between KKP and host proteins (e.g., MvaU) or the Pf4 core protein RepG4 (*40*, *41*), we now also reveal cross-talk involving accessory TA genes of the Pf4 and Pf6 prophages. This complex, dynamic interplay further underscores the critical role of prophage-encoded TA systems in establishing phage-host and phage-phage symbiosis within biofilm communities. Given the high diversity of Pf phage accessory genomes, enriched with TA genes (*61*), these TAs are likely critical mediators of prophage-prophage and prophage-host dynamics. Targeting these prophage-encoded TAs therefore presents a promising strategy against recalcitrant *P. aeruginosa* infections. The underlying approach would be to selectively activate toxin expression (e.g., PfkA/PfkB and PfiT), thereby repressing Pf induction during biofilm formation. Disrupting this process would reduce biofilm integrity and potentiate antibiotic efficacy (*40*), offering a path to alternative combination therapies.

ParDE systems represent one of the earliest identified TA families. While all known ParE toxins belong to a single Protein Families Database (PFAM) family (PF05016), their cognate antitoxins are associated with multiple PFAM families (*62*). Structural analyses of ParDE complexes and ParE-associated antitoxins have provided insights into toxin activation and TA system physiology. ParE-containing TA systems predominantly adopt binary architectures, with ternary variants also documented (*44*, *63*). These two-component systems generally assemble into complexes with antitoxin-toxin stoichiometries of 2:2 (*38*, *43*, *50*), 4:2 (*38*), or 6:2 (*51*). In ternary systems, the antitoxin’s N terminus lacks DNA binding capability, necessitating an additional regulatory protein from the gene cluster for transcriptional repression and can attain an 8:8 stoichiometric ratio (PDB 5CW7 and 5CZE) (*44*). In this study, comparative structural analysis of the ParDE family identified two unique functional domains: the C-terminal extension of the PfiT toxin and the N-terminal PAD domain of the PfiA antitoxin. However, the PfiAT complex exhibits a noncanonical 6:4 assembly. Crucially, crystal structure analysis reveals that this unique PfiA_6_PfiT_4_ configuration depends on the structural features of the PfiT C-terminal extension combined with the N-terminal PAD domain of PfiA. Furthermore, these distinct stoichiometries influence both toxin release and autoregulation of the TA operon. The formation of the canonical 2:2 TA complex reduces DNA binding affinity, resulting in up-regulation of toxin gene expression.

Recent studies have shown that some type II antitoxins bound to their cognate toxins exhibit increased resistance to protease degradation compared to the free antitoxins (*64*–*66*). Although PfiA has a disordered C terminus, which might render it susceptible to proteolysis similar to the PrpA antitoxin of the plasmid-encoded ParDE TA system PrpTA (*62*) and MqsA (*39*, *67*), it becomes stabilized upon complex formation with PfiT. However, the PfiAT system uses a distinct strategy for toxin activation involving posttranslational modification. Two recent independent studies revealed that antitoxins in the *M. tuberculosis* VapBC (*68*) and RelJK (*69*) type II TA systems are phosphorylated by intracellular Ser/Thr kinases PknA and PknK, respectively. Phosphorylation at specific sites within the antitoxin’s toxin-binding domain weakens its interaction with the toxin, leading to toxin release. Structural evidence demonstrates that phosphorylation of T5 in PfiA introduces a negative charge, disrupting hydrophobic interactions that stabilize adjacent dimers. This triggers a conformational change that converts the complex from a 6:4 to a 2:2 stoichiometry, resulting in loss of DNA binding ability. Consistent with this mechanism, phosphomimetic mutations in type II VapB antitoxins severely impair DNA binding to cognate promoter sequences (*68*). Thus, such posttranslational modifications represent a potentially widespread regulatory strategy in TA systems, warranting further investigation.

## METHODS

### Plasmid constructions

The information of strains and plasmids used in this study is detailed in table S2. The WT full-length *pfiT* and *pfiA* genes were amplified by polymerase chain reaction (PCR) from genomic DNA from PAO1 strain using the primers *pfiT*-F/R and *pfiA*-F/R, respectively. After double digestion with the corresponding restriction enzymes, the two genes were sequentially ligated into the multiple cloning site (MCS) of the pETDuet-1 vector (Merck). After *pfiT* was inserted into MCS1 using BamH I and Hind III restriction sites, *pfiA* was inserted into MCS2 of the same vector using Nde I and Xho I sites. To efficiently cleave off the N-terminal His-tag, the PreScission protease cleavage site was inserted between 8 × His-tag and the MCS1. For pMQ70-based constructs, different fragments were amplified with primer pairs in table S3. After digested with Kpn I and Hind III, the fragments were ligated into pMQ70 empty vector digested with the same two enzymes. The mutants of the two genes were generated by the QuikChange method (Stratagene) using the WT as the template. In the phosphorylation assay, the *pfkA*-*pfkB*-*pfpC* system gene was cloned into the pHERD20T vector, while the *pfiA* gene was constructed into the pET28a (+) vector. Both vectors were digested with Nco I and Hind III (for pHERD20T) or Nde I and Xho I (for pET28a), followed by ligation of the corresponding fragments.

### Toxicity assays

The *pfiA* and *pfiT* genes, along with the *pfiAT* complex and their truncated mutants, were cloned into the pMQ70 plasmid. The recombinant plasmids were transformed into *E. coli* WM3064 and then conjugated into *P. aeruginosa* MPAO1. Toxicity was assessed using growth curve analysis and plate streaking assays. Single colonies of MPAO1 carrying the recombinant plasmids were inoculated into 3 ml of LB medium supplemented with ampicillin (100 μg ml^−1^; Amp) and grown at 37°C to log phase. For growth curves, cultures were diluted to OD_600_ (optical density 600 nm) = 0.05 in fresh LB containing 5 mM l-arabinose (Ara) and Amp (100 μg ml^−1^). Two hundred microliters of aliquots was transferred to 96-well plates, and growth was monitored at 37°C using a microbial growth analyzer (VIEWKR). For the plate streaking assay, log-phase cultures were streaked onto LB agar plates supplemented with either 5 mM Ara and Amp (100 μg ml^−1^; test) or Amp only (100 μg ml^−1^; control). Plates were incubated at 37°C for 12 hours, and growth was visually assessed and photographed.

### Protein expression and purification

The expression plasmids of pETDuet-1/*pfiT*/*pfiA* or mutants were transformed into *E. coli* strain BL21 (DE3) cells for overexpression of the target proteins. The transformed cells were cultured overnight in LB broth containing Amp (100 μg ml^−1^) at 37°C. A 500 ml of fresh culture medium was inoculated with 5 ml of overnight culture. When the OD_600_ value reached 0.6 to 0.8 at 37°C, the expression of the target proteins was induced by 0.3 mM isopropyl-β-d-thiogalactopyranoside (IPTG), and the culture was kept shaking overnight at 25°C. The cells were then pelleted by centrifugation at 3030*g* for 15 min and resuspended in prechilled nickel–nitrilotriacetic acid (Ni-NTA) buffer A containing 40 mM tris-HCl (pH 8.0), 250 mM NaCl, 10 mM imidazole, 1 mM β-mercaptoethanol (β-ME), and 1 mM phenylmethylsulfonyl fluoride (PMSF). The resuspended cells were lysed by ultrasonication, and the supernatant was obtained by centrifugation at 23,500*g* for 60 min at 4°C. The supernatant was then applied onto Ni-NTA affinity resin (QIAGEN) preequilibrated with Ni-NTA buffer A. The target protein was eluted with Ni-NTA buffer B containing 40 mM tris-HCl (pH 8.0), 250 mM NaCl, 250 mM imidazole, 1 mM β-ME, and 1 mM PMSF. The 8 × His-tag at the N terminus was cleaved off by being treated with protease overnight at 4°C in the presence of 5 mM β-ME, which was subsequently applied onto a Histrap column (Cytiva) to remove uncut protein. The unbound portion was pooled and dialyzed against a buffer containing 20 mM tris-HCl (pH 8.0), 150 mM NaCl, and 1 mM dithiothreitol (DTT). The protein was further concentrated to 2.5 mg ml^−1^, flash cooled in liquid nitrogen, and stored at −80°C.

### Chromosomal gene knockout in MPAO1

A previously described method was adapted and modified to construct deletion mutants in MPAO1. Primers were designed to amplify the upstream and downstream homologous arms (1000 to 2000 bp each) flanking the target gene. The homologous arms were then fused with the modified suicide vector pEX18Gm using a one-step cloning method to facilitate homologous recombination. The recombinant plasmid was introduced into *E. coli* WM3064–competent cells via chemical transformation. After PCR verification, *E. coli* WM3064 harboring the correct recombinant plasmid was used as the donor strain for conjugation. The donor strain and the recipient MPAO1 strain were cultured and mixed at a ratio of 2:1 (donor:recipient). The mixture was spotted onto LB agar plates supplemented with 1% diaminopimelic acid and incubated at 30°C for 6 to 12 hours to allow conjugal transfer. In-frame deletion mutants were generated through homologous recombination and selected using the sucrose counter-selection method. The mutations were confirmed by PCR and DNA sequencing using the primer pair gene-LF/LR.

### Flow-cell biofilm assay

Biofilm formation was analyzed using a flow cell system as previously described with minor modifications (*40*). Briefly, sterile silicone catheters were inoculated with 1 ml of overnight bacterial culture and allowed to adhere for 1 hour under static conditions. Continuous flow of modified M9 medium was then maintained at 0.1 ml min^−1^ using a peristaltic pump. Biofilm samples were collected at designated time points by aseptically opening the catheters, while effluent was monitored for phage release. The medium was refreshed daily, and experiments were conducted over 6 to 8 days at room temperature.

### Phage production and plaque assay

Flow cell effluents from MPAO1 WT, Δ*pfiT*, and *pfiT*^1–100^ at different culture days were collected, and phages were separated using a 0.22-μm micropore filter. The MPAO1 and MPAO1 ΔPf4ΔPf6 cultures, grown to an OD_600_ of approximately 1.0, were then mixed with molten R-Top medium in a 1:3 ratio and overlaid on LB agar plates. After spotting 5 μl of 10-fold serially diluted phage solution onto the top, the double-layer plates were incubated overnight at 37°C following absorption of the liquid droplets.

### MALDI-TOF mass spectrometry

The sample for analysis by MALDI mass spectrometry is prepared by mixing or coating with solution of an energy-absorbent, organic compound called matrix. When the matrix crystallizes on drying, the sample entrapped within the matrix also cocrystallizes. The sample within the matrix is ionized in an automated mode with a laser beam. Desorption and ionization with the laser beam generate singly protonated ions from analytes in the sample. The protonated ions are then accelerated at a fixed potential, where these separate from each other based on their mass/charge ratio (*m/z*). The charged analytes are then detected and measured using different types of mass analyzers like quadrupole mass analyzers, ion trap analyzers, time-of-flight (TOF) analyzers, etc. For microbiological applications, mainly TOF mass analyzers are used. During MALDI-TOF analysis, the *m/z* ratio of an ion is measured by determining the time required for it to travel the length of the flight tube.

### Size exclusion chromatography coupled to right-angle light scattering

The molecular weight in solution of samples was measured using SEC-RALS. The SEC-RALS system was equilibrated with buffer A [150 mM NaCl and 20 mM tris-HCl (pH 8.0)] at 0.5 ml min^−1^ for 2 hours before the sample loading. One hundred microliters of protein samples (2.5 mg ml^−1^) in the same buffer was centrifuged at 23,500*g* for 10 min at 4°C and then injected into the analytical Superdex 200 Increase 10/300 GL column separating and detecting by the AKTA Purifier System (Cytiva, USA) coupled with a RALS instrument (DAWN HELEOS 8; Wyatt Technologies, USA) with a flow ratio of 0.5 ml min^−1^. Average weight molecular mass of each sample was determined on the basis of the data processed by the ASTRA (v7.0.1) offered by Wyatt Company.

### Crystallization, data collection, and structure determination

The crystal screens were set up at room temperature using the sitting-drop vapor diffusion method in 96-well plates. The sample of PfiT and PfiA complex was centrifuged at 23,500*g* for 10 min at 4°C before crystallization. The final concentration of the proteins was 2.5 mg ml^−1^. Initial screens were manually set up using the PEGRx and SaltRx and Index screens (Hampton Research, USA). Hits were observed 3 days later, and rod-shaped crystals were crystallized under a well solution containing 22% polyethylene glycol monomethyl ether (PEG MME) 550, 0.1 M DL-Malic acid (pH 7.0), and 0.1 M imidazole. All crystals were cryoprotected by soaking in a solution of the reservoir condition plus 20% glycerol (v/v).

Native diffraction data of PfiAT were collected using the beamline 19U1 (BL19U1) at the Shanghai Synchrotron Radiation Facility and was processed with the program HKL3000 (*70*). The structure was solved by molecular replacement using the program PHENIX (*71*) with the AlphaFold 3–predicted structures of ap-form PfiT and PfiA as the search model. The initial models generated by molecular replacement for the PfiT-PfiA cocrystals contain a PfiA dimer and two PfiT monomers in the asymmetric unit. The other two PfiA dimers and two PfiT monomers in the model were manually built by Coot (*72*) according to the electron density map. Then, the rebuilt model was fed to the refinement program phenix. refine (*73*), and multiple cycles of refinement were conducted, followed by model rebuilding. The final model was validated by MolProbity (*74*). All data collection and refinement statistics are presented in table S1.

### Structure prediction

The structural models of PfiT and the PfiAT-DNA complex were predicted using AlphaFold 3 (*75*). Structural homology was assessed using the Dali server, and molecular graphics were generated with ChimeraX1.9 (www.cgl.ucsf.edu/chimerax) (*76*, *77*).

### Electrophoretic mobility shift assay

The DNA fragments of different lengths were diluted with TE buffer [10 mM Tris-HCl, 1 mM ethylenediaminetetraacetic acid (EDTA), pH 8.0] to 200 μM, and then the forward and reverse primers were mixed in equal volumes. The mixture was heated at 75°C for 5 min and cooled to room temperature for annealing to form double-stranded DNA. Proteins were incubated with DNA at different molar ratios in 10 μl of buffer containing 100 mM NaCl, 20 mM tris-HCl (pH 8.0), and 1 mM DTT on ice for 30 min. The reaction sample was then mixed with 10 μl of 0.5× tris-boric acid–EDTA (TBE) buffer (pH 8.3) containing 30% glycerol, and 20 μl of the sample was loaded onto a 6% polyacrylamide gel separately. Electrophoresis was performed at 4°C for 60 min at 100 V after prerunning the gel for 45 min, with 0.5× TBE buffer as the running buffer. The gels were stained with ethidium bromide.

### Microscopic observation

Overnight cultures of MPAO1 carrying the empty pMQ70, pMQ70-*pfiT*, and A12 mutants’ plasmids were diluted with LB with carbenicillin (100 μg ml^−1^) at an OD_600_ of 0.1, and 1 mM Ara was added at OD_600_ of ~0.5 to induce the expression of proteins. Cells were collected at 2 hours by centrifugation at 3000*g* for 5 min and then stained with 4′,6-diamidino-2-phenylindole (1 μg ml^−1^) and propidium iodide (1 μg ml^−1^) for 10 min and subsequently imaged.

### Phosphoproteomic analysis

PfiA-His was expressed in *E. coli* BL21 (DE3) using the IPTG-inducible pET28a (+) vector, while different components of the KKP system were expressed using the Ara-inducible pHERD20T vector in *E. coli* and MPAO1. Given that PfkA/PfkB tends to form inclusion bodies and exhibits low enzymatic activity when overexpressed, a dual-plasmid system was used to coexpress KKP and PfiA. Cultures at the logarithmic growth phase (OD_600_ = 0.6 to 0.8) were cooled to 16°C, and KKP expression was induced with 10 mM Ara for 2 hours, followed by induction of PfiA-His with 0.1 mM IPTG for 4 hours. Cells were harvested by centrifugation at 6000*g* for 5 min at 4°C. The cell pellets were resuspended in lysis buffer [150 mM NaCl, 20 mM tris-HCl (pH 8.0), 5 mM β-ME, 1% protease inhibitor cocktail, and 1% phosphatase inhibitor] and sonicated on ice at 220 W (5 s on, 5 s off) for 5 min. Cell debris was removed by centrifugation at 12,000*g* for 30 min at 4°C. PfiA-His protein was purified using Ni-NTA affinity chromatography. Protein concentration was determined using Bradford reagent (Bio-Rad). For sample preparation, 3 μg of target protein was separated by SDS-PAGE, and the protein band of PfiA was excised with a clean blade. Procedures for tryptic digestion, phosphopeptide enrichment, liquid chromatography–tandem mass spectrometry (MS/MS), and data analysis were performed as previously described (*40*, *78*, *79*). Briefly, the obtained protein was digested in trypsin solution at a protein-to-protease ratio of 50:1 (m/m) for 12 hours. Subsequently, 5 mM DTT was added, and the mixture was incubated at 56°C for 30 min, followed by the addition of 11 mM iodoacetamide and incubation in the dark at room temperature for 15 min. The digested peptides were dissolved in enrichment buffer containing 50% acetonitrile. Modified peptides were extracted and vacuum-dried with 10% ammonia. The resulting peptides were then subjected to tandem MS/MS coupled with ultra-performance liquid chromatography (UPLC) on a Q Exactive Plus instrument. The acquired MS/MS data were processed using the MaxQuant search engine (v.1.6.15.0) and searched against the MPAO1 or *E. coli* BL21 (DE3) proteome for peptide identification. The mass spectrometric analysis of phosphorylated peptides is summarized in tables S4 and S5.

### β-Galactosidase activity assay

The promoter activity was assessed by measuring the β-galactosidase activity using a modified Miller assay (*42*, *80*). The experiments used MPAO1Δ*pfiAT* strains carrying the *pfiA* promoter–*lacZ* fusion construct.
