# Supplementary material for: Phosphorylation of PfiA modulates Pf4 phage production through PfiA/PfiT stoichiometric reconfiguration in Pseudomonas aeruginosa
Source: Sci Adv. 2026 Apr 3;12(14):eaeb5480. doi: 10.1126/sciadv.aeb5480 (PMC13048266; doi:10.1126/sciadv.aeb5480)
Supplement: Supplementary file 1 — Figs. S1 to S8 Tables S1 to S5 [file sciadv.aeb5480_sm.pdf]

Science Advances
AAAS

Supplementary Materials for

Phosphorylation of PfiA modulates Pf4 phage production through PfiA/PfiT stoichiometric reconfiguration in *Pseudomonas aeruginosa*

Ran Chen *et al.*

Corresponding author: Xiaoxue Wang, xxwang@scsio.ac.cn

Sci. Adv. **12**, eaeb5480 (2026)
DOI: 10.1126/sciadv.aeb5480

This PDF file includes:

Figs. S1 to S8
Tables S1 to S5

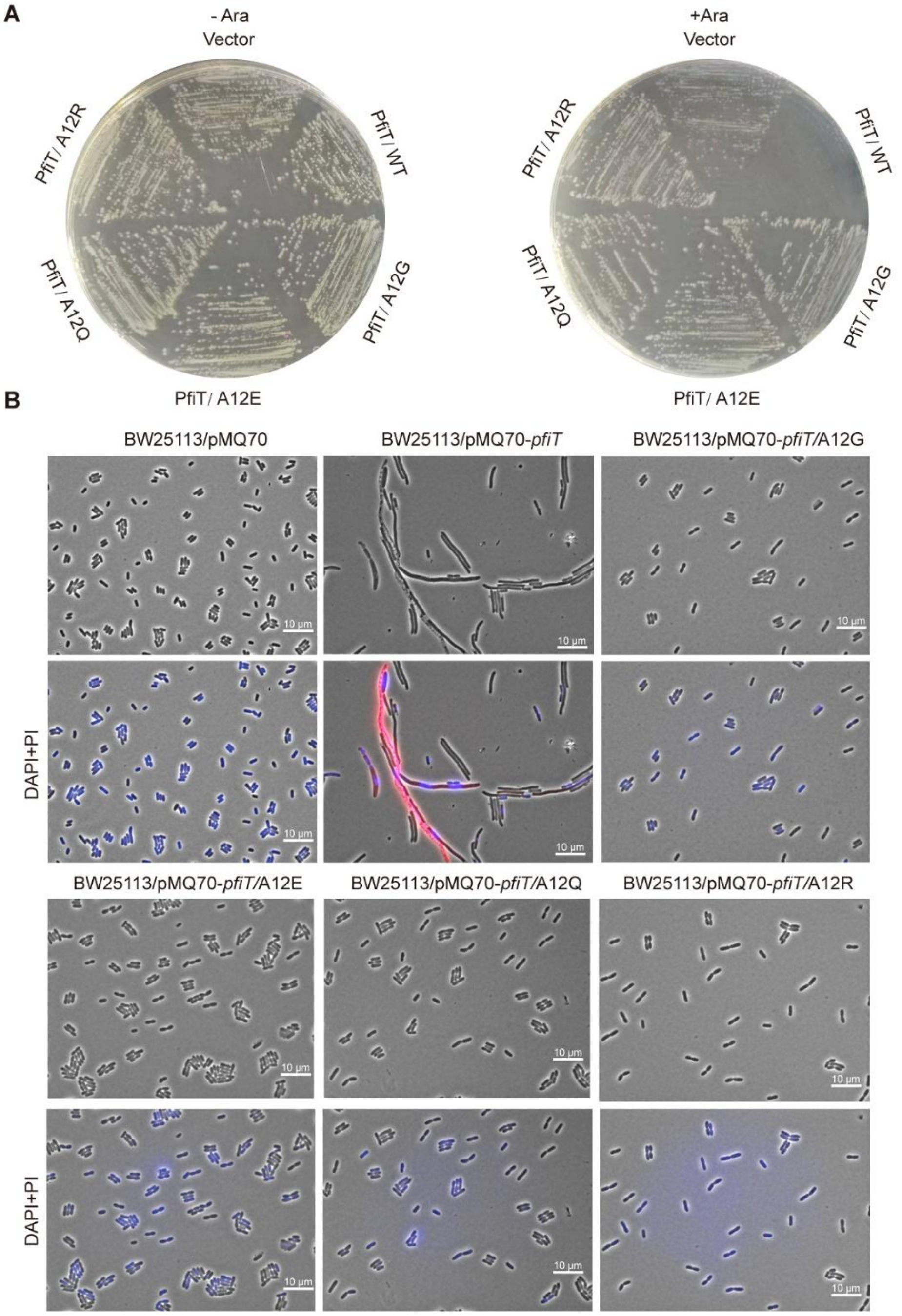

Fig. S1. The A12 residue is crucial for the stability of PfiT. (**A**) Toxicity assays comparing wild-type and A12-mutated PfiT. (**B**) Microscopy of MPAO1 cells after induction with 10 mM arabinose for 2 h. Three independent replicates were performed, and representative images are shown here.

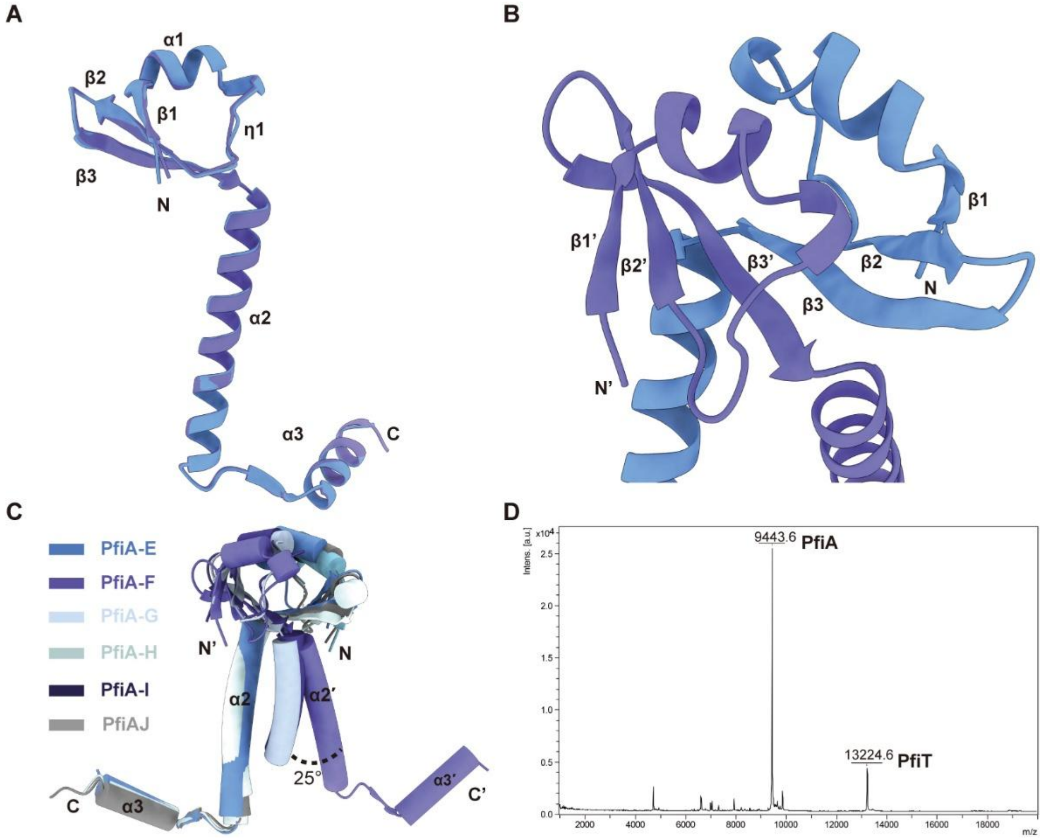

Fig. S2. Characterization and molecular weight determination of PfiA in the complex. (**A**) Superimposition of the two monomers of PfiA/chain E and chain F homodimer. The secondary structure elements and the N- and C-terminal are indicated. (**B**) The six β-strands of two PfiA monomers forms a β-sheet. (**C**) A superposition of the three PfiA dimers in PfiAT complex. The color of each chain has been labeled on the left side. (**D**) MALDI-TOF mass spectrometry identifies the molecular weight of proteins in the PfiAT crystal sample.

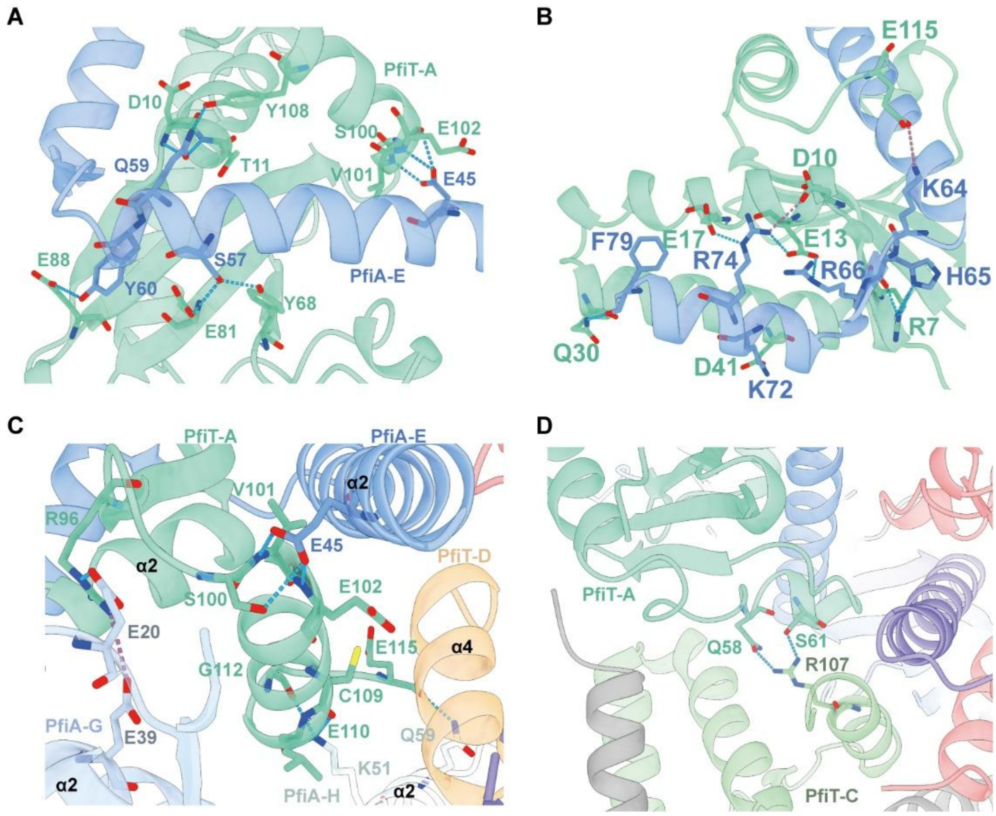

Fig. S3. Key interactions involved in the assembly of the PfiAT complex. (**A-B**) Interaction between PfiT/chain A and the bound PfiA/chain E. (**C-D**) Hydrogen bonds formed by PfiT/chain A C-terminus (six pairs) and α3 region (two pairs).

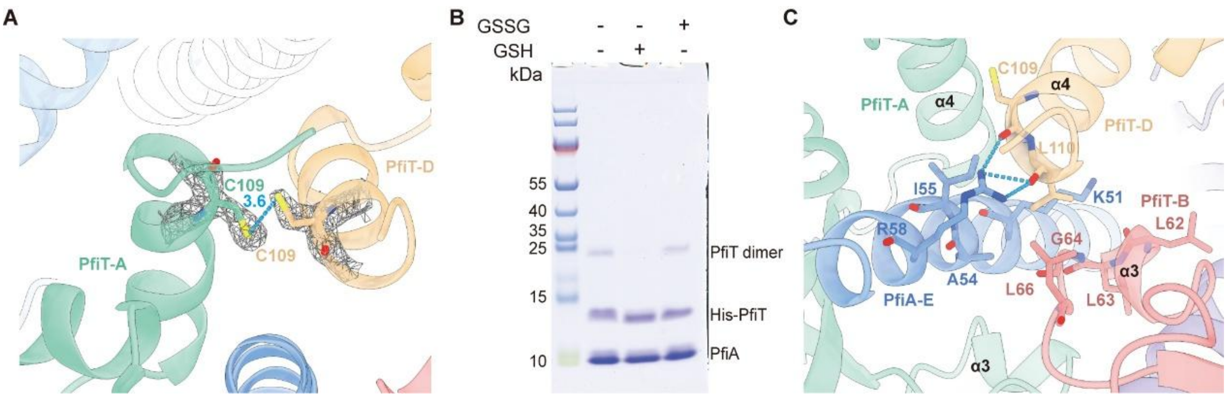

Fig. S4. Interactions between PfiT subunits stabilize the quaternary structure. (**A**) The spatially localization of two cysteine residues between PfiT/chains A and D. The composite omit map of the two cysteine was indicated by the gray mesh. (**B**) Analysis of disulfide bond-mediated PfiT dimerization under redox conditions by SDS-PAGE. GSSG and GSH served as the oxidizing and reducing agents, respectively, in the protein buffer. (**C**) Interaction involving the additional α2 of PfiT/chain A compared to homologous proteins.

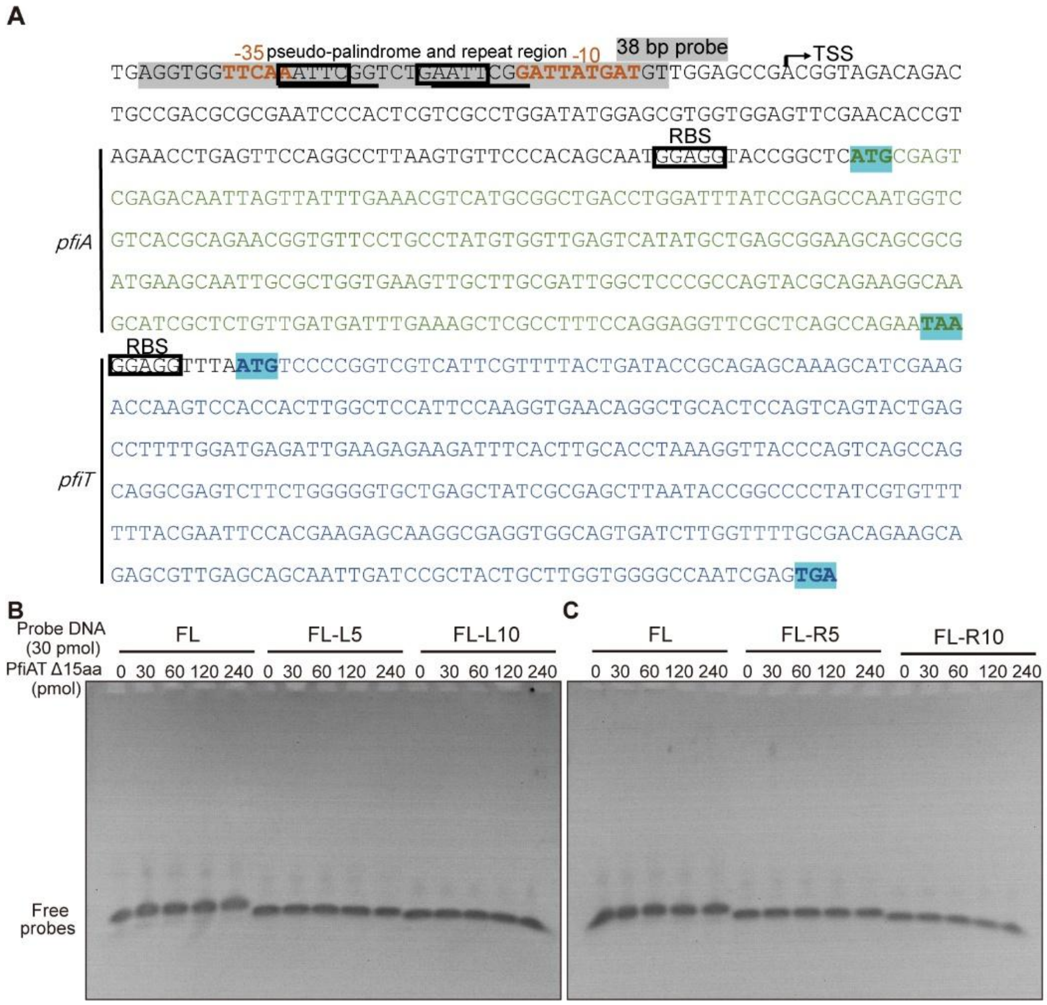

Fig. S5. C-terminal truncation of PfiT completely abolishes the DNA-binding activity of the PfiAT complex. (**A**) Sequence of *pfiAT* operon within prophage Pf4. The antitoxin *pfiA* is shown by the green letters, while the toxin *pfiT* is shown by the blue letters. The sequence analysis of the *pfiAT* operon is indicated. The -10 and -35 regions are shown in orange letters. The transcriptional start site (TSS) and RBS are also shown. The 38bp-FL DNA sequence is highlighted in grey, with boxed regions representing pseudo-palindromic sequences, and underlined regions denoting repeated sequences. (**B**-**C**) The EMSA assay on the binding affinities of the mutant complex concerning the C-terminal in PfiT. The molar quantities of the protein and DNA are shown above the gel while bands for the free and protein-bound DNA complex are indicated on the left.

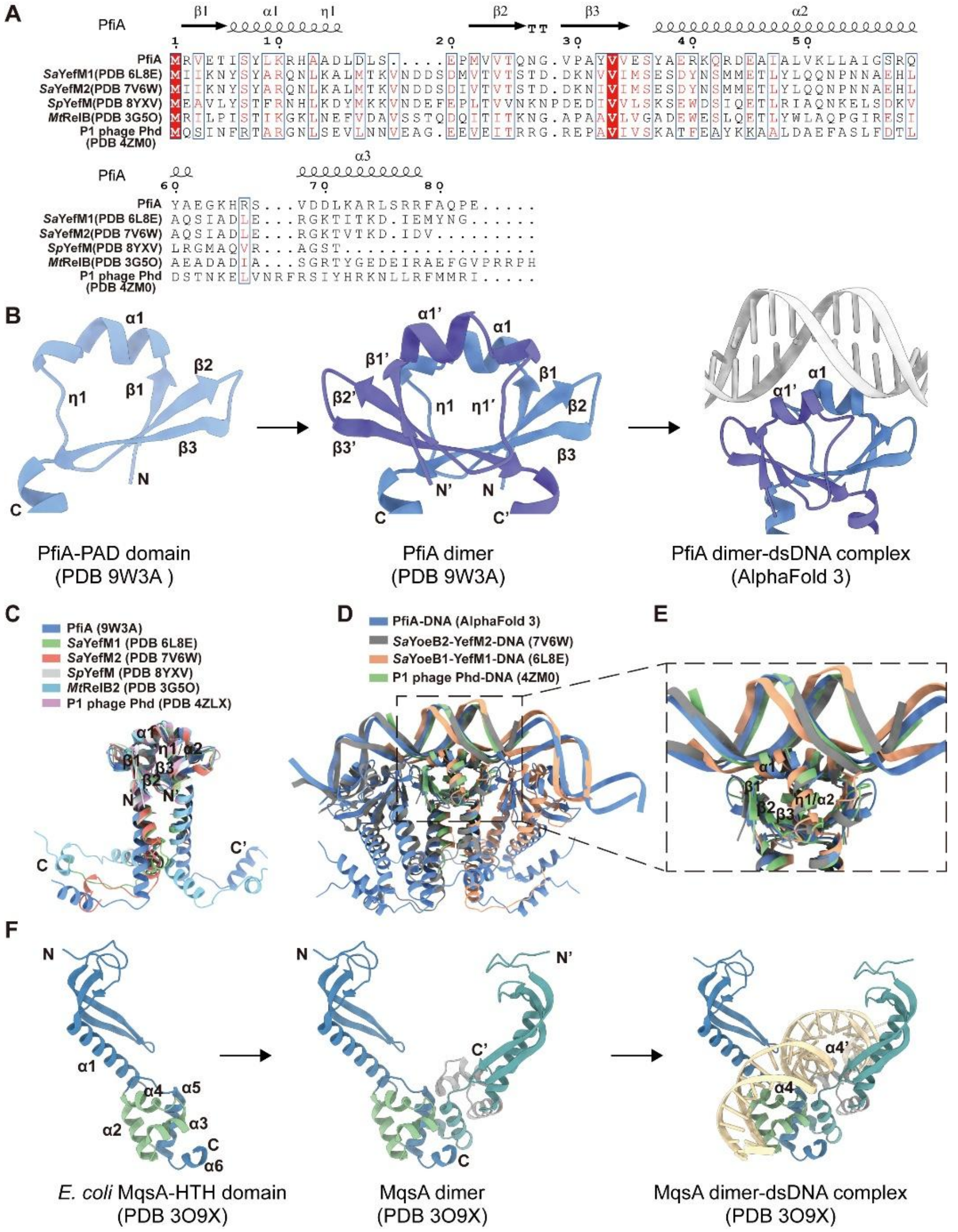

Fig. S6. Several antitoxins, including RelB, YefM, and Phd, share PAD domain with PfiA. (**A**) Multiple sequence alignment of PfiA with orthologs and other published structures. The secondary structure elements were labeled above the sequences. (**B**) The N-terminal structures of PfiA monomers and dimers, along with the AlphaFold 3-predicted complex model of their interaction with DNA. The α1 helix of PfiA is inserted into the major groove of the DNA double helix. (**C**) Structural superimposition of the backbone structure of PfiA dimer (colored blue) onto that of *Sa*YefM1 (PDB 6L8E) and *Sa*YefM2 (PDB 7V6W) from *S. aureus*, *Sp*YefM from *S. pneumoniae* (PDB 8YXV), *Mt*RelB2 from *M. tuberculosis* (PDB 3G5O) and P1 phage Phd (PDB 4ZLX). (**D-E**) Two views of the structural superposition between the PfiA-DNA complex from AlphaFold 3 and homologous PAD domain-DNA complexes. (**F**) The monomeric, dimeric, and DNA-bound complex structures of *E. coli* MqsA antitoxin (PDB 3O9X). The α4 helix of MqsA inserts into the major groove of the DNA double helix. The three α helices that constitute the HTH domain are shown in green and gray.

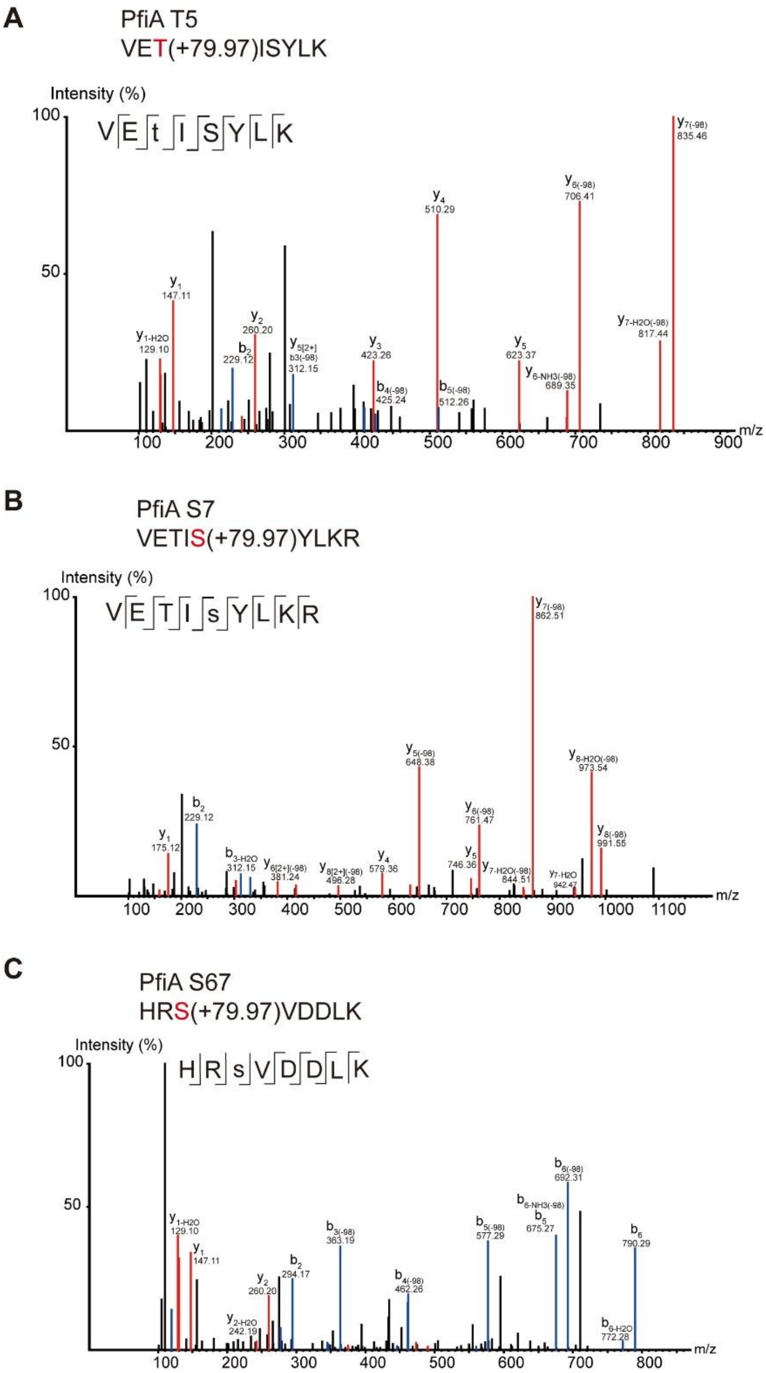

Fig. S7. Characteristic LC-MS/MS spectra of identified phosphopeptides containing the T5 modification.

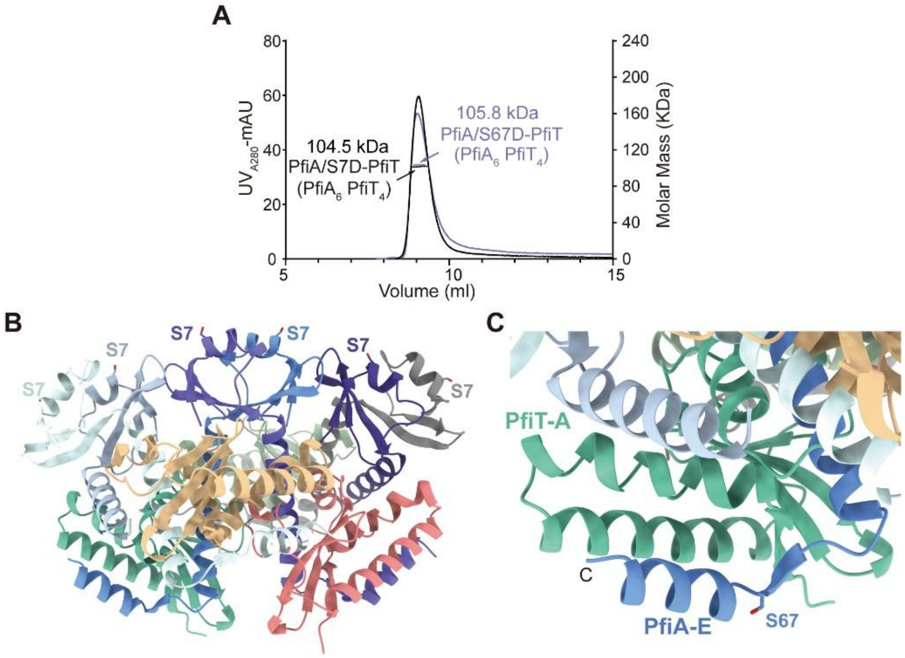

Fig. S8. Phosphorylation at S7 and S67 does not alter the PfiA: PfiT stoichiometry. (**A**) SEC-RALS analysis of the molecular weight of the PfiA/S7D/S67D-PfiT. (**B-C**) Localization of S7 and S67 within the PfiA structure.

Table S1. Data collection and refinement statistics.

	9W3A
**Data collection**	
Space group	$P2_12_12_1$
Cell dimensions	
a, *b*, *c* (Å)	73.17, 107.4, 125.72
α, β, γ (°)	90, 90, 90
R_{merge} (%)	49.39-2.3 (2.382-2.3)
Rpim (%)	0.09174 (0.6941)
$CC_{1/2}$ (%)	
$I / \sigma I$	26.12 (3.70)
Completeness (%)	99.41 (99.23)
Redundancy	13.3 (13.2)
**Refinement**	
Resolution (Å)	49.39-2.3 (2.382-2.3)
No. reflections	44497 (4378)
R_{work} / R_{free}	20.24 / 25.00
No. atoms	7129
Protein	6964
Ligand/ion	0
Water	165
Average B-factor (Å^2)	48.25
Protein	48.33
Ligand/ion	0
Water	44.81
R.m.s. deviations	
Bond lengths (Å)	0.008
Bond angles (°)	0.96
Ramachandran plot (%) (favored/allowed/outliers)	98.36/1.64/0.00

Table S2. Bacterial strains and plasmids used in this study. R indicates resistance, Gm indicates gentamycin, Amp indicates ampicillin, Car indicates Carbapenems, and Km indicates kanamycin.

	Description	Source
**Strains**		
MPAO1	A widely-distributed PAO1 derivative	lab stored
MP-ΔPf4ΔPf6	whole Pf4 and Pf6 prophage removed from MPAO1 host chromosome	lab stored
MPAO1*pfiT*$^{1\text{-}100}$	C-terminal extension of the *pfiT* gene removed from the MPAO1 host chromosome	this study
MPAO1Δ*pfiA*Δ*pfiT*::*pfiA*^{promoter}-*lacZ*	*pfiA* and *pfiT* genes were deleted from the MPAO1 chromosome and replaced with a *lacZ* reporter gene.	lab stored
MPAO1Δ*pfiT*	*pfiT* gene removed from the MPAO1 host chromosome	lab stored
Top10	F^-, *mcrA*Δ(*mrr-hsd RMS-mcrBC*), *φ80, lacZΔM15*, Δ*lacX74, recA1, araΔ139Δ(ara-leu)7697, galU, galK, rps*, (*Strr*) *endA1, nupG*	lab stored
BL21 (DE3)	F^-*ompT hsdS*$_B$(*r*$_B^-$*m*$_B^-$) *gal dcm* *λ*(DE3) Ω P_{tacUV5}::T7 *polymerase*	Novagen
WM3064	*thrB*1004 *pro thi rpsL hsdS lacZ*ΔM15 RP4-360 Δ(*araBAD*)567 Δ*dapA*1341::[*erm pir*]	W. Metcalf, UIUC
BTH101	*F-, cya-99, araD139, galE15, galK16, rpsL1 (Strr), hsdR2, mcrA1, mcrB1*	Euromedex Kit
**Plasmids**		
pEX18Gm	GmR, *oriT*+, *sacB*+, gene replacement vector	lab stored
pEX18Gm-*pfiT*$^{1\text{-}100}$ up down	Gm^R, Ap^R, for deleting C-terminal extension of the *pfiT* gene	this study
pMQ70	Car^R, Ap^R, expression vector	lab stored
pMQ70-*pfiA-pfiT*	Car^R, *pfiA-pfiT* was cloned into pMQ70	this study
pMQ70-*pfiA*	Car^R, *pfiA* was cloned into pMQ70	this study
pMQ70-*pfiT*	Car^R, *pfiT* was cloned into pMQ70	this study
pMQ70-*pfiT*$^{1\text{-}100}$	Car^R, *pfiT*$^{1\text{-}100}$ was cloned into pMQ70	this study
pMQ70-*pfiA-pfiT*$^{1\text{-}100}$	Car^R, *pfiA-pfiT*$^{1\text{-}100}$ was cloned into pMQ70	this study
pETDuet	Ap^R, expression vector	lab stored
pETDuet-*pfiA-pfiT*	Ap^R, *pfiA* in Pf4 was cloned into upstream, *pfiT* cloned into downstream	this study

pETDuet-*pfiA*-*pfiT*$^{1\text{-}100}$	Ap^{R}, *pfiA* in Pf4 was cloned into downstream, *pfiT*$^{1\text{-}100}$ cloned into downstream.	this study
pHERD20T	Ap^{R}, expression vector with araC-P$_{BAD}$ promoter	lab stored
pHERD20T-*pfkA*-*pfkB*	Ap^{R}, pfkA-*pfkB* in pHERD20T *Nco* I/*Hind* III	this study
pHERD20T-*pfkA*-*pfkB*-*pfpC*	Ap^{R}, pfkA-*pfkB*-*pfpC* in Pf6 were cloned into pHERD20T	this study
pET28a (+)	Km^{R}, expression vector with T7 promoter	lab stored
pET28a-*pfiA*	Km^{R}, *pfiA* in Pf4 was cloned into pET28a	this study

Table S3. Oligonucleotides used in this study. F indicates forward primer and R indicates reverse primer. The red letters indicate the mutation of nucleotides.

Primer name	Sequence (5'-3')	Purpose
pMQ70-*pfiA*-*pfiT*-F	TTTTTTGGGCTAGCGAATTCGAGCTCGGTACCGGCTCATGCGAGTCGAGACAATTAGTT	toxicity assay
pMQ70-*pfiA*-*pfiT*-R	CATCCGCCAAAACAGCCAAGCTTCTACTCGATTGGCCCCACCAAGC	
pMQ70-*pfiA*-F	TTTTTTGGGCTAGCGAATTCGAGCTCGGTACCGGCTCATGCGAGTCGAGACAATTAGTT	
pMQ70-*pfiA*-R	TCCGCCAAAACAGCCAAGCTTTCAGTGGTGGTGGTGGTGGTGTTCTGGCTGAGCGAACC	
pMQ70-*pfiT*-F	TTTTTTGGGCTAGCGAATTCGAGCTCGGTACCGGCTCATGTCCCCGGTCGTCATTCGTT	
pMQ70-*pfiT*-R	CATCCGCCAAAACAGCCAAGCTTCTACTCGATTGGCCCCACCAAGC	
pMQ70-*pfiT*$^{1-100}$-F	TTTTTTGGGCTAGCGAATTCGAGCTCGGTACCGGCTCATGTCCCCGGTCGTCATTCGTT	
pMQ70-*pfiT*$^{1-100}$-R	ATCTTCTCTCATCCGCCAAAACAGCCAAGCTTCTAGCTCTGCTTCTGTCGCAAAACCAA	
pMQ70-*pfiA*-*pfiT*$^{1-100}$-F	TTTTTTGGGCTAGCGAATTCGAGCTCGGTACCGGCTCATGCGAGTCGAGACAATTAGTT	
pMQ70-*pfiA*-*pfiT*$^{1-100}$-R	ATCTTCTCTCATCCGCCAAAACAGCCAAGCTTCTAGCTCTGCTTCTGTCGCAAAACCAA	
pMQ70-*pfiT*-A12E-F	CATTCGTTTTACTGATACCGAAGAGCAAAGCATCGAAGAC	
pMQ70-*pfiT*-A12E-R	GTCTTCGATGCTTTGCTCTTCGGTATCAGTAAAACGAATG	phosphorylation assay
pMQ70-*pfiT*-A12Q-F	CATTCGTTTTACTGATACCCAAGAGCAAAGCATCGAAGAC	
pMQ70-*pfiT*-A12Q-R	GTCTTCGATGCTTTGCTCTTGGGTATCAGTAAAACGAATG	
pMQ70-*pfiT*-A12R-F	CATTCGTTTTACTGATACCAGAGAGCAAAGCATCGAAGAC	
pMQ70-*pfiT*-A12R-R	GTCTTCGATGCTTTGCTCTCTGGTATCAGTAAAACGAATG	
pHERD20T-*pfkB*-R	ACGACGGCCAGTGCCAAGCTTTCATAAGTTTATCTCCGGATGAGATAG	
pHERD20T-*pfpC*-F	AGGAGATATACATACCCATGAATGTGAACTTAGAGAACGATATTACATTTC	
pHERD20T-*pfpC*-R	ACGACGGCCAGTGCCAAGCTTCTAGTCATCCTTGATGGGGAAAGA	
pETDuet-upstream-*pfiA*-F	TAAGAAGGAGATATACCATGCGAGTCGAGACAATTAGTT	Construction of pETDuet-*pfiA*-*pfiT* and related single site mutations in *pfiA*
pETDuet-upstream-*pfiA*-R	GCATTATGCGGCCGCAAGCTTTATTCTGGCTGAGCGAACC	
pETDuet-downstream-*pfiT*-F	GAAGGAGATATACATATGGCAATGTCCCCGGTCGTCATTCG	
pETDuet-downstream-*pfiT*-R	GGTTTCTTTACCAGACTCGATCAGTGGTGATGATGGTGATGCTCGATTGGCCCCACC	

pETDuet-downstream-*pfiT*$^{1\text{-}100}$-R	GGTTTCTTTACCAGACTCGATCAGTGGTGATGATGGTGATGGCTCTGCTTCTGTCGCAA	
Duet-*pfiA*-T5D-F	TACAATGCGAGTCGAGGACATTAGTTATTTGAAA	
Duet-*pfiA*-T5D-R	GTTTCAAATAACTAATGTCCTCGACTCGCATTGTA	
Duet-*pfiA*-T5A-F	TACAATGCGAGTCGAGGCAATTAGTTATTTGAAA	
Duet-*pfiA*-T5A-R	GTTTCAAATAACTAATTGCCTCGACTCGCATTGTA	
Duet-*pfiA*-S7D-F	GCGAGTCGAGACAATTGATTATTTGAAACGTCATG	
Duet-*pfiA*-S7D-R	CATGACGTTTCAAATAATCAATTGTCTCGACTCGC	
Duet-*pfiA*-S67D-F	CAGAAGGCAAGCATCGCGATGTTGATGATTTGAAAGCTC	
Duet-*pfiA*-S67D-R	GAGCTTTCAAATCATCAACATCGCGATGCTTGCCTTCTG	
Duet-PfiA-K10A-F	GAGACAATTAGTTATTTGGCACGTCATGCGGCTGACCTG	
Duet-PfiA-K10A-R	CAGGTCAGCCGCATGACGTGCCAAATAACTAATTGTCTC	
Duet-PfiA-R11A-F	ACAATTAGTTATTTGAAAGCTCATGCGGCTGACCTGGAT	
Duet-PfiA-R11A-R	ATCCAGGTCAGCCGCATGAGCTTTCAAATAACTAATTGT	
Duet-*pfiA*-N27A-F	CAATGGTCGTCACGCAGGCCGGTGTTCCTGCCTAT	
Duet-*pfiA*-N27A-R	CATAGGCAGGAACACCGGCCTGCGTGACGACCAT	
Duet-*pfiA*-G28A-F	TGGTCGTCACGCAGAACGCTGTTCCTGCCTATGTG	
Duet-*pfiA*-G28A-R	CCACATAGGCAGGAACAGCGTTCTGCGTGACGAC	
Duet-*pfiA*-P30A-F	GTCACGCAGAACGGTGTTGCTGCCTATGTGGTTG	
Duet-*pfiA*-P30A-R	CTCAACCACATAGGCAGCAACACCGTTCTGCGTG	
pfiAT-promoter-38bp-F	AGGTGGTTCAAATTCGGTCTGAATTCGGATTATGATGT	EMSA assay
pfiAT-promoter-38bp-R	ACATCATAATCCGAATTCAGACCGAATTTGAACCACCT	
pfiAT-promoter-38bp-L5-F	GTTCAAATTCGGTCTGAATTCGGATTATGATGT	
pfiAT-promoter-38bp-L5-R	ACATCATAATCCGAATTCAGACCGAATTTGAAC	
pfiAT-promoter-38bp-L10-F	AATTCGGTCTGAATTCGGATTATGATGT	
pfiAT-promoter-38bp-L10-R	ACATCATAATCCGAATTCAGACCGAATT	

pfiAT-promoter-38bp-R5-F	AGGTGGTTCAAATTCGGTCTGAATTCGGATTAT	
pfiAT-promoter-38bp-R5-R	ATAATCCGAATTCAGACCGAATTTGAACCACCT	
pfiAT-promoter-38bp-R10-F	AGGTGGTTCAAATTCGGTCTGAATTCGG	
pfiAT-promoter-38bp-R10R	CCGAATTCAGACCGAATTTGAACCACCT	
pEx18ap-*pfiT*-delC-up-F	ACGACGGCCAGTGCCAAGCTTTTCGGATTATGATGTTGGAG	
pEx18ap-*pfiT*$^{\triangle 101-115}$-up-R	TTTTCTTGTTATCGCAATAGTCAGCTCTGCTTCTGTCGCA	Construction of
pEx18ap-*pfiT*$^{\triangle 101-115}$-Gm-F	TGCGACAGAAGCAGAGCTGACTATTGCGATAACAAGAAAA	pEX18Gm-*pfiT*$^{1-100}$::Gm
pEx18ap-*pfiT*$^{\triangle 101-115}$-Gm-R	ATGCTCAGGAGTAGAAAGCCATTAGGTGGCGGTACTTGGGT	up down
pEx18ap-pfiT$^{\triangle 101-115}$-dn-F	ACCCAAGTACCGCCACCTAATGGCTTTCTACTCCTGAGCAT	
pEx18ap-*pfiT*$^{\triangle 101-115}$-dn-R	TATGACCATGATTACGAATTCACATCTACACCACGCCCCAC	
pEx18ap-*pfiT*$^{\triangle 101-115}$-LF	TGACCCTACAGCACATCCTG	Verification of strain
pEx18ap-*pfiT*$^{\triangle 101-115}$-LR	ACGAGCACGAATACCACCAG	MPAO1/*pfiT*$^{1-100}$

Table S4. LC-MS/MS analysis identified phosphopeptides of PfiA in Flow cell samples.

Modification sites information													
Protein accession	Position	Amino acid	Gene name	Localization probability	PEP	Score	Charge	Modified sequence	Mass error [ppm]	MS/MS Counts	Flow cell sample_1	Flow cell sample_2	Flow cell sample_3
PA1_4488	5	T	pfiA	0.999856	0.011399	101.82	2	VET(1)ISYLK	-0.70379	4	0.645892207	0.588465737	1.765642056

Table S5. LC-MS/MS analysis detected phosphorylated PfiA peptides in BL21(DE3) harboring the dual-plasmid expression system.

Protein	Protein Description	Protein Position	Modified Peptide Sequence Window	Modifications	Best -10logP	Best Ion Intensity (%)	Best Ion Intensity (%) Sample 1	Sample 1 modified	Sample 1 unmodified
E. coli BL21 (DE3)/pET28a-*pfiA* and pHERD20T									
protein1|PfiA	user pasted sequence	S7	****MRVETIsYLKRHAADLD	Phosphorylation (STY)	64.91	6	6	3.62E+06	2.15E+10
protein1|PfiA	user pasted sequence	S19	LKRHAADLDLsEPMVVTQNGV	Phosphorylation (STY)	76.92	3	3	2.00E+07	6.64E+10
E. coli BL21 (DE3)/pET28a-*pfiA* and pHERD20T-*pfkA-pfkB*									
protein1|PfiA	user pasted sequence	T5	******MRVEtISYLKRHAAD	Phosphorylation (STY)	79.86	7	7	1.36E+08	3.38E+10
protein1|PfiA	user pasted sequence	S7	****MRVETIsYLKRHAADLD	Phosphorylation (STY)	68.64	6	6	1.97E+07	3.43E+10
protein1|PfiA	user pasted sequence	S67	SRQYAEGKHRsVDDLKARLSR	Phosphorylation (STY)	44.63	16	16	5.67E+06	3.06E+08
E. coli BL21 (DE3)/pET28a-*pfiA* and pHERD20T-*pfkA-pfkB-pfpC*									
protein1|PfiA	user pasted sequence	S7	****MRVETIsYLKRHAADLD	Phosphorylation (STY)	70.52	4	4	5.94E+06	2.55E+10
protein1|PfiA	user pasted sequence	S67	SRQYAEGKHRsVDDLKARLSR	Phosphorylation (STY)	49.35	18	18	1.20E+06	8.73E+07
